# Hydrodynamically decoupled nanoprecipitation (HDNP): A multi-stage strategy for supersaturation, controlled delayed nucleation, and stabilization of low LogP poorly-water soluble drugs^☆^

**DOI:** 10.1016/j.ijpx.2026.100656

**Published:** 2026-08-30

**Authors:** Luis Castillo-Henríquez, Clélia Mathieu, Rabah Gahoual, Dhanang Edy Pratama, Cyrille Richard, Nathalie Mignet, José Roberto Vega-Baudrit, Yohann Corvis

**Affiliations:** a Université Paris Cité, CNRS, INSERM, UTCBS, Unité de Technologies Chimiques et Biologiques pour la Santé, F-75006 Paris, France; b Laboratory of Pharmaceutical Physical Chemistry, Faculty of Pharmacy, University of Costa Rica, San José 11501-2060, Costa Rica; c National Nanotechnology Laboratory (LANOTEC), National Center for High Technology (CeNAT-CONARE), San José 1174-1200, Costa Rica; d Department of Chemical and Materials Engineering, National Central University, Taoyuan City 320317, Taiwan

**Keywords:** Amorphous nanoparticles, Nanocrystals, Hydrodynamics, CIJ mixer, Pulsatile flow, Microfluidics, Solvent-antisolvent nanoprecipitation, Computational fluid dynamics

## Abstract

Poor aqueous solubility is a major drawback of modern drug candidates, affecting their biopharmaceutical performance. Solvent-antisolvent methods involving rapid mixing, such as Flash Nanoprecipitation and Sequential Nanoprecipitation, have advanced the field. However, these remain restricted to compounds with relatively high hydrophobicity (minimum LogP ≥4.5–6). To address this limitation, we introduce Hydrodynamically Decoupled Nanoprecipitation (HDNP). The approach enables decoupling supersaturation, nucleation, and stabilization by controlling the hydrodynamic environments. Dexamethasone (DEX, LogP: 1.83) was used as a model drug. A mechanistically and physics-informed framework was developed, integrating in-line dynamic light scattering for count rate evolution assessment, Computational Fluid Dynamics (CFD), and Design of Experiments for design space establishment. CFD revealed periodic shear stress, high turbulent dissipation rates, and local flow reversal during pulsatile flow under various Womersley numbers, providing favorable hydrodynamic conditions for controlled delayed nucleation. HDNP of DEX successfully produced mainly amorphous nanoparticles (DEX-NPs) stabilized with P407. DEX-NPs exhibited a hydrodynamic diameter of 241 nm, PdI of 0.246, loading efficiency of 77%, enhanced dissolution relative to the micronized drug, and physicochemical stability for at least two weeks. The approach was further evaluated using prednisolone (LogP: 1.62). These findings demonstrate the applicability of HDNP to the nanoprecipitation of the used corticosteroids and highlights its potential as a promising platform for other poorly water-soluble compounds with low LogP.

## Highlights

• HDNP enables the nanoprecipitation of molecules with LogP ˂ 2.
• Pulsatile shear and hydrodynamic fluctuation control delayed induced nucleation.
• HDNP enhances nucleation and stabilization timescales for low LogP drugs.
• Increase in Womersley number during pulsatile flow enhances hydrodynamic diameter.
• HDNP-P407-based preparations exhibited superior drug retention and stability.

## Graphical abstract

**Figure:**
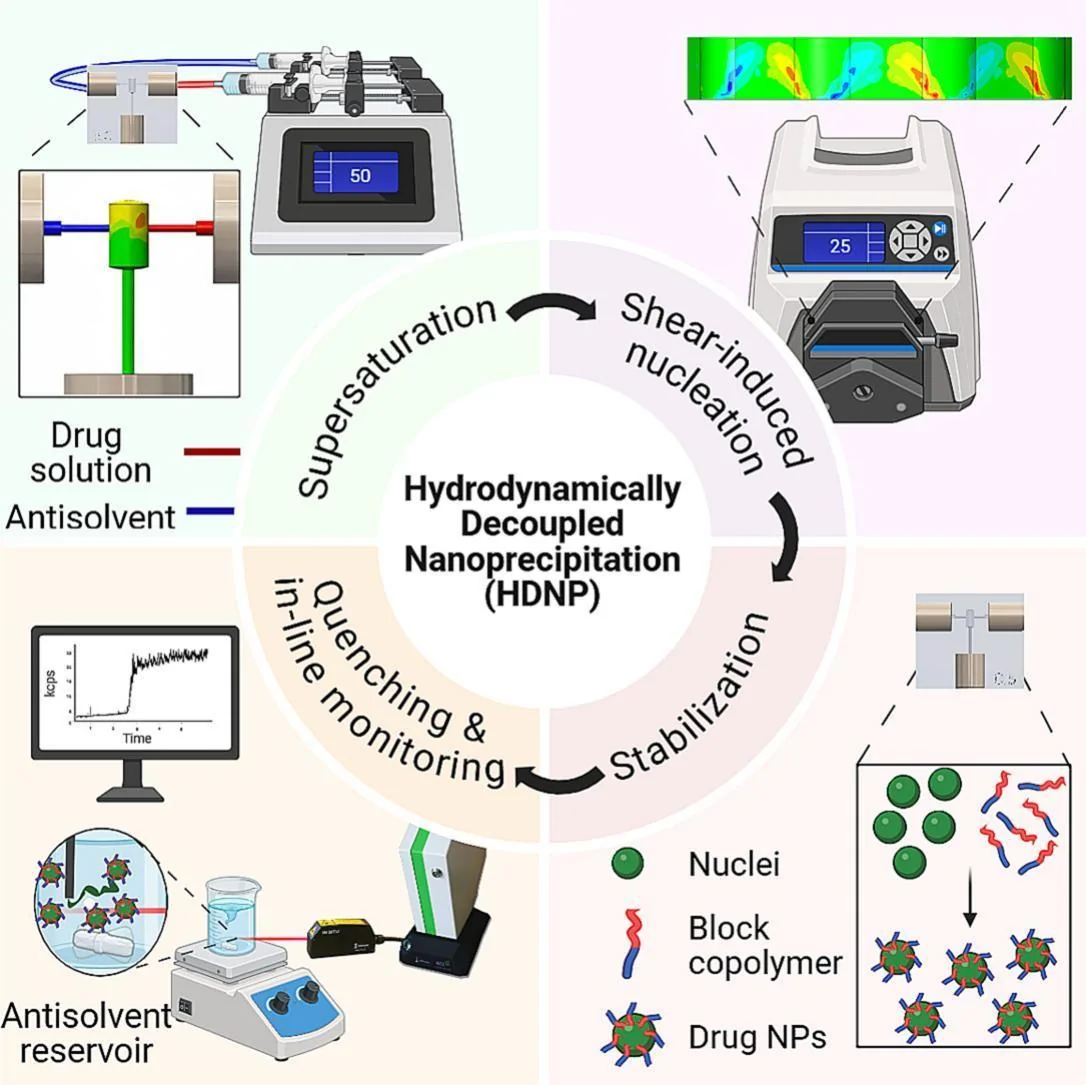

## 1 Introduction

Poor aqueous solubility remains one of the primary challenges limiting the biopharmaceutical performance of modern drug candidates (Nyamba et al., 2024). In this context, solvent-antisolvent nanoprecipitation is a versatile and scalable bottom-up approach for producing amorphous or crystalline nanoparticles (NPs), enabling an enhanced dissolution profile. The obtention of either solid forms depends on the formulation and the operational parameters within the designed process (Jermain et al., 2018). In pharmaceutical applications, an organic solvent containing a poorly water-soluble drug is mixed with an antisolvent (i.e., water). This leads to decreased solubility and the generation of supersaturation, which drives nucleation and particle growth (Castillo Henríquez et al., 2024).

Despite the apparent simplicity of the nanoprecipitation protocols, achieving consistent particle size and a low polydispersity index (PdI) requires precise control over supersaturation, which varies both temporally and spatially (Saad and Prud'homme, 2016). To address this challenge, rapid mixing strategies have been developed to achieve high supersaturation rates (Johnson and Prud'homme, 2003a; Liu et al., 2008; Han et al., 2012; Caggiano et al., 2023). In this regard, Johnson and Prud'homme pioneered the Flash Nanoprecipitation (FNP) of active organic compounds in 2003 (Johnson and Prud'homme, 2003a). A drug solution and an antisolvent — usually containing an amphiphilic block-copolymer (BCP) or surfactant — are fed into opposing inlets of a confined impinging jet (CIJ) mixer. Remarkably, the technique enables extremely rapid mixing on the millisecond timescale.

More recently, in 2023, Caggiano et al. (Caggiano et al., 2023) proposed Sequential Flash Nanoprecipitation (SNaP), building on prior knowledge from FNP to further optimize the formulation of stable core-shell NPs. This method allows for decoupling nucleation and stabilization by using two CIJs in series. Therefore, nucleation and growth of the hydrophobic core take place at the first mixing unit, whereas stabilization with the BCP occurs in the second mixer. The inter-mixer delay time (*T*_*d*_) between core formation and stabilization was further explored by Belinky et al. (Belinky et al., 2025) through a modular 3D-printed millifluid platform. The precise control on this parameter enabled demonstrating the tunable growth of drug-loaded polymeric particles from the nanometer to the micrometer scale.

Several hydrophobic active pharmaceutical ingredients (APIs) have been successfully nanoprecipitated employing the previous techniques, such as paclitaxel (Zhu et al., 2010), curcumin (Chow et al., 2015), and itraconazole (Wan et al., 2018). However, despite the advances experienced over the last 20 years, from classical FNP to SNaP, fast precipitation in the CIJ chamber is mostly affected by the molecule's LogP value (Ristroph et al., 2025; Tao et al., 2019). Successful nucleation and growth before stabilization with a BCP has been generally reported to be feasible for active compounds with a minimum LogP ranging between 4.5 (Pagels et al., 2018) and 6 (Pustulka et al., 2013), otherwise drug NPs are prone to Ostwald ripening and recrystallization (Pustulka et al., 2013).

The most critical cases are encountered for APIs with LogP ˂ 2 (low positive LogP), where, despite their classification as poorly water-soluble drugs, the aqueous solubility is too high, and nucleation kinetics are not adequately addressed by rapid mixing alone (Zhu, 2014). For such systems, the generated supersaturation is often insufficient to overcome the nucleation energy barrier within the characteristic short residence time (*t*_*r*_) for FNP-based methods (Saad and Prud'homme, 2016). Notably, these compounds may also undergo non-classical nucleation pathways, involving the formation of metastable intermediates (e.g., prenucleation clusters) (Sleutel and Van Driessche, 2014). Therefore, a shift towards strategies that enable controlling the temporal evolution of supersaturation and the induction of delayed nucleation under defined hydrodynamic conditions is required for low LogP compounds.

**Fig. 1:**
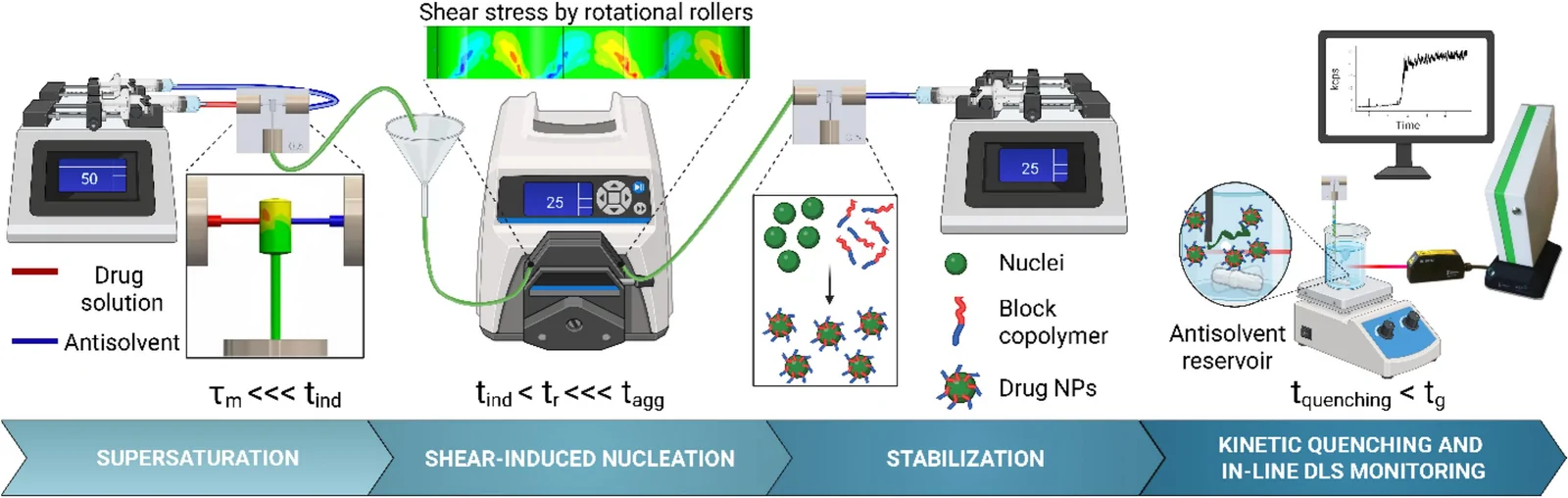
Hydrodynamically Decoupled Nanoprecipitation (HDNP) workflow: supersaturation is generated by mixing the solvent and antisolvent at equal flow rates (FRs) in a CIJ unit. The supersaturated solution is discharged into a funnel that is connected to the inlet of an elastomer tubing in a peristaltic pump. The funnel enables adjusting the FR according to the operational capacity of the peristaltic pump, while also avoids non-desired additional suction. Pulsatile flow induces nucleation by hydrodynamic shear fluctuations. Stabilization takes place in a second CIJ unit, where the nuclei suspension is mixed with the aqueous solution of the block copolymer (i.e., stabilizing agent), which adsorbs onto the nuclei surface. The nanosuspension is finally discharged into an antisolvent reservoir for growth quenching and count rate monitoring by in-line DLS.

In this context, we introduce Hydrodynamically Decoupled Nanoprecipitation (HDNP). The approach enables decoupling supersaturation, nucleation, and stabilization by controlling the hydrodynamic environment (Fig. 1). The initial step involves CIJ mixing of the organic solution and the antisolvent in absence of the stabilizing agent (Caggiano et al., 2023). Under these conditions, the characteristic mixing time (*τ*_*m*_) is shorter than the induction time (*t*_*ind*_, i.e., the detectable onset of rapid spontaneous nucleation). Unlike in FNP and SNaP, this stage results only in a metastable supersaturated solution without immediate nucleation due to the compound's hydrophobicity (Devos et al., 2021).

The main novelty of the approach lies in the induced nucleation under the combined influence of pulsatile shear and hydrodynamic perturbations, resulting from flowing through a peristaltic pump. For this purpose, the setup is minimally disconnected from the supersaturation stage using a funnel to allow changing the flow rate (FR) and avoid additional undesired suction by the peristaltic pump. The latter feature enables increasing *t*_*r*_ long enough for API core formation and growth without aggregation (*t*_*agg*_) before the second CIJ unit. There, stabilization is achieved by the addition of a BCP, injected at the same FR of the nuclei suspension, to adsorb onto the formed nuclei. Finally, kinetic quenching (*t*_*quenching*_) is produced by effluent discharging into an antisolvent reservoir to minimize NPs growth (*t*_*g*_), ultimately enabling the obtention of a stable nanosuspension.

Notably, the HDNP temporal decoupling strategy differs fundamentally from the previously mentioned SNaP approaches. The latter primarily controls the growth of the cores from the first rapid mixing step by changing *T*_*d*_ before stabilization in the second mixer (Belinky et al., 2025). Conversely, HDNP introduces a new level of temporal control by initially decoupling supersaturation from nucleation. Hydrodynamic perturbations due to pulsatile shear therefore control the onset of the delayed nucleation. The distinction between the two nanoprecipitation approaches is particularly relevant for poorly water-soluble drugs with low LogP, especially considering the advantages in terms of *t*_*r*_ provided by HDNP, which are required to promote their nucleation and core growth.

Therefore, this work provides a new nanoprecipitation platform for challenging APIs. Specifically, dexamethasone (DEX, *S*_*0*_: 0.0096 mg mL^−1^ at 25 °C) (Islam and Mitra, 2022) was used as a model drug given its LogP of 1.83 (Ita, 2020). DEX controlled delayed nucleation was assessed as a function of the Womersley number (*α*). A mechanistically and physics-informed framework was also developed by integrating computational fluid dynamics (CFD) and in-line dynamic light scattering (DLS). HDNP process resulted predominantly in amorphous NPs, with NCs being formed to a lesser extent following a systematic Design of Experiments (DoE) approach. The protocol was successfully applied for prednisolone (PRD, LogP: 1.62) (PubChem, 2026) nanoprecipitation as well. Notably, the approach demonstrated control over the critical quality attributes (CQAs) under flow-based conditions, thereby meeting current demands for automation and continuous manufacturing.

## 2 Materials and methods

### 2.1 Materials

DEX (CAS No. 50–02-2, MW: 392.46 g mol^−1^, 99% purity) — donated by EuroAPI (Vertolaye, France) — and PRD (CAS No. 50–24-8, MW: 360.44 g mol^−1^, 99% purity) — obtained from Sigma-Aldrich (*St*. Louis, MO, USA) — were used without further purification. Absolute anhydrous ethanol (CAS No. 64–17-5) and methanol HPLC grade (CAS No. 64–56-1) were purchased from Carlo Erba (Val de Reuil, France). Kolliphor P407 and P188 (Poloxamer 407 and P188, CAS 9003-11-6) — amphiphilic tri-BCPs composed of polypropylene oxide (PPO) and polyethylene oxide (PEO) — were provided by Basf (Ludwigshafen, Germany). Phosphate-Buffered saline (PBS) was provided by Thermo Fisher Scientific (Waltham, MA, USA). Photopolymer resin ABS-like Pro 2 — mainly composed of acrylate/urethane acrylate oligomers, reactive monomers, photo-initiators, and other additives — was purchased from Anycubic (Shenzhen, China). Ultrapure water was obtained using a Merck Milli-Q system (Darmstadt, Germany), which filtered distilled water through two ion-exchange.

### 2.2 Computer-aided design and 3D printing

**Fig. 2:**
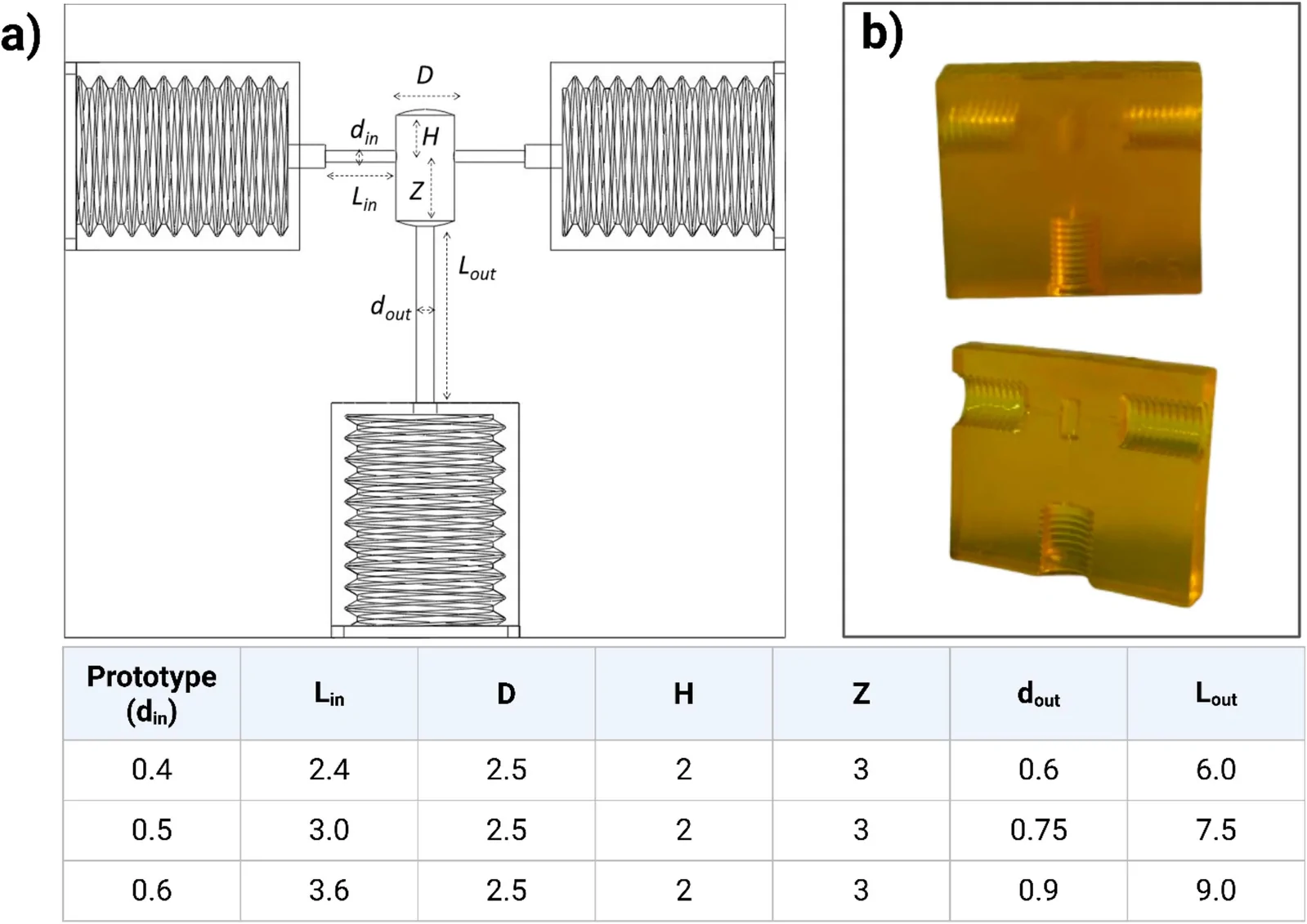
CIJ mixer: basic dimensional configuration of the three prototypes employed based on the inlet internal diameter (*d*_*in*_) **(a)**. The inset table shows the corresponding dimensions (mm) for *d*_*in*_, inlet pipe length (*L*_*in*_), chamber diameter (*D*), height from impinging plane (*H*), length from impinging plane to outlet pipe (*Z*), outlet diameter (*d*_*out*_) and outlet pipe length (*L*_*out*_). Representative 3D-printed unit (top) with a vertical cross-sectional view (bottom) **(b)**. The modifiable stl. File of the designed CIJ unit is available at https://github.com/luiscasth94/CIJ-mixer.git

3D modeling of CIJ units was done using the CAD software SolidWorks (v.2024, Dassault Systems, France). The basic dimensional configuration is a modified version inspired on the model proposed by Johnson and Prud'homme (Johnson and Prud'homme, 2003b) (cf. SI). The dimensions of the three prototypes used throughout the work are presented in Fig. 2. No additional inlet connectors were included as the model has proven adequate printability and resin drainage, as well as ease of cleaning between experiments. The 3D models were sliced in Photon Workshop (v3.6.0, Shenzhen, China) and printed by stereolithography (SLA) using a Photon Mono M7 Pro 3D printer (Shenzhen, China) and the ABS-like resin Pro 2 at a controlled temperature (30 °C) by the vat heating system. Printed units were washed with isopropanol for 5 min. Post-print UV light curing (405 nm) was done using an Anycubic Wash & Cure 3 (Shenzhen, China) for 5 min at room temperature.

### 2.3 Physicochemical characterization

#### 2.3.1 DEX nucleation and P407 self-assembling assessment

Nucleation and stabilization timescales assessment was performed by in-line monitoring of scattered intensity during stirring. The VascoKin particle size analyzer (Cordouan Technologies, France) in-situ probe (λ: 658 nm; scattering angle: 170°) was used to assess the count rate (kcps) evolution upon effluent discharging. A one-minute recording pre-discharging was performed for all experiments involving an antisolvent reservoir to set a baseline. When required, a milli-fluidic probe was used accordingly.

#### 2.3.2 Hydrodynamic diameter, polydispersity, particle concentration, and zeta potential

HD and PdI of the obtained systems were evaluated by DLS based on their translation diffusion coefficient at 25 °C, using the Stokes-Einstein equation. A 1 mL sample was transferred into a ZEN0040 cuvette at predetermined time points and analyzed using a Malvern Zetasizer Ultra (Worcestershire, UK) (λ = 633 nm, scattering angle: 173°, and laser position: 3 mm). Concentration of the suspensions in terms of particles per mL was determined by multi-angle DLS (MADLS) using a DTS0012 cuvette. Zeta potential (ζP) was determined using the Smoluchowski equation. The samples were loaded into a DTS1070 cuvette with 10 mM NaCl, and the electrophoretic mobility was measured at 25 °C.

#### 2.3.3 Transmission electron microscopy and cryogenic method

Transmission electron microscopy (TEM) was performed using a 200-mesh carbon formvar copper grid, covered with 10 μL of the suspension. Drying was done for two hours following blotting. For cryogenic-TEM (cryo-TEM), samples were prepared with an EM-GP2 cryoplunger (Leica Microsystems, France). A 4 μL drop of the suspension was deposited onto a glow-discharged 300-mesh copper grid coated with a lacey carbon film. After blotting for five seconds, the grid was rapidly vitrified by plunging into liquid ethane cooled to −180 °C. Later, the grids were mounted on an Elsa cryo-holder (Ametek SAS, France) and examined at −170 °C. The analyses were performed using a Jeol 1400 transmission electron microscope (Jeol, France) operating at 120 kV and fitted with a RIO CMOS camera (Ametek SAS, France). Particle sizes were determined using Image J (NIH, USA, v.1.54p).

#### 2.3.4 DEX quantification and drug loading efficiency

DEX content was quantified by HPLC using a Shimadzu DGU-20A3R HPLC (Kyoto, Japan) and C18 (5 μm, 250 × 4.6 mm) Nucleodur® column (Düren, Germany). The mobile phase consisted of a 65:35 *v*/v methanol:water solution with an isocratic flow at 1.0 mL min^−1^. The limit of quantification (LOQ) and limit of detection (LOD) were calculated as performed by Concenza et al. (Urban et al., 2009), yielding values of 0.418 and 0.138 μg mL^−1^, respectively. A 0.5 mL sample of the DEX NPs was completely dissolved in 1.5 mL of methanol by sonication for 30 min and filtered using a Merck Millex 0.22 μm polyethersulfone (PES) membrane filter (Darmstadt, Germany) to remove the polymer. A 20 μL sample of the filtered solution was injected, and the drug content was determined using a calibration curve performed from 1 to 100 μg mL^−1^ at 239 nm (r^2^ = 1). For evaluation over time, the result was expressed as API content (%) relative to the initial drug concentration using **Eq. 1**, with *C*_*t*_ being the concentration at time *t* and *C*_*0*_ the DEX concentration at *t*_*0*_.

(1) $$\text{Potency}\%=\frac{C_{t}}{C_{0}}*100$$

Additionally, loaded DEX was quantified indirectly by filtration of the nanosuspensions using the 0.22 μm PES filters. A 20 μL filtered solution of the solubilized free drug was analyzed following the HPLC protocol. The loading efficiency (LE%) was determined by **Eq. 2**, where *m*_*t*_ is the total mass of the drug (mg), and *m*_*f*_ is the mass (mg) of free drug.

(2) $$\mathrm{LE}\%=\frac{m_{t}-m_{f}}{m_{t}}*100\;$$

#### 2.3.5 Drug release kinetics

In vitro release of DEX from the nanosuspensions was evaluated using a Slide-A-Lyzer Mini Dialysis device with a 10 kDa MWCO cellulose membrane (Thermo Scientific, Rockford, IL, USA). As a control, micronized DEX was dispersed with the stabilizing agent at the same DEX concentration and BCP:DEX ratio. The dispersions were subjected to sonication for 1 h before the evaluation. A 1.5 mL aliquot of each sample was transferred into the 2 mL device. Subsequently, the device was immersed in a conical tube containing 44.5 mL of PBS as the release medium reservoir. Sink conditions were established following the criterion proposed by Lombardo et al. (Lombardo et al., 2021), using a reservoir volume of the medium at least 3–5 times the volume required to reach drug saturation at a defined temperature (*V*_*sat*_). In this case, the *V*_*sat*_ at 37.5 °C for the maximum possible DEX amount (∼375 μg) was 5.82 mL.

The complete release setup was left inside an Innova 43 incubator (Eppendorf New Brunswick, Montesson, France) at 37.5 °C and 130 rpm for 24 h. Aliquots were withdrawn from the reservoir compartment at 1, 3, 6, 10, and 24 h and transferred to a Greiner 384-well microplate (Kremsmünster, Austria). An equivalent volume of fresh PBS at the same temperature was added after each sampling point to maintain a constant reservoir volume. Samples were analyzed by UV–Vis spectroscopy using a TECAN Spark, and the concentration was quantified by a calibration curve performed from 1 to 50 μg mL^−1^ (r^2^: 0.9998). Cumulative release percentage data were fitted to a non-linear regression for an exponential plateau, expressed in **Eq. 3**:

(3) $$Y=Y_{M}-\left(Y_{M}-Y_{0}\right)*e^{-\mathrm{kx}}$$

where *Y* is the cumulative release percentage at a given time *x* (h), *Y*_*0*_ is the starting released percentage, *Y*_*M*_ represents the maximum attainable release (i.e., plateau), and *k* is the rate constant expressed in h^−1^.

### 2.4 Hydrodynamically decoupled nanoprecipitation

#### 2.4.1 Proof-of-concept

The low LogP poorly-water soluble drug was dissolved in ethanol at 5 mg mL^−1^. A net volume of two mL of this organic solution was mixed against the same net volume of an antisolvent stream in a CIJ unit of 0.5 mm inlet diameter, controlled by a Harvard PHD ultra-syringe pump apparatus (Holliston, MA, USA) at 50 mL min^−1^ per stream. The effluent was directly discharged into a funnel (4 cm stem length with 3 mm diameter) connected to the inlet of a Tygon A-60-G (Saint-Gobain, Courbevoie, France) thermoplastic elastomer tubing (23 cm length, 3.2 mm internal diameter) in a Gilson Minipuls3 peristaltic pump (Middleton, WI, USA). The equipment was set at the maximum angular velocity (47.9 rpm) to avoid stagnation, resulting in a FR of 25 mL min^−1^. Subsequently, the intermediate effluent was mixed in a second CIJ unit with a net volume of 4 mL of a 5 or 10 mg mL^−1^ P407 aqueous solution (P407:DEX 2:1 or 4:1 mass ratio, respectively) at an equal FR to the peristaltic pump, controlled by a second syringe pump apparatus. The final effluent was then discharged into an antisolvent reservoir volume according to the dilution factor to be tested, and stirred at 500 rpm for 15 min.

#### 2.4.2 Womersley number assessment in pulsatile flow

The Womersley number (*α*) was used to assess variations in the pulsatile flow regime as defined in **Eq. 4**:

(4) $$\alpha =R\sqrt{\frac{\omega \rho }{\mu }}$$

where *R* is the tube radius (m), *ρ* is the fluid density and *μ* the dynamic viscosity, with the last two assumed as 900 kg m^−3^ and 2.5*10^−3^ Pa s, respectively, for a 50:50 ethanol:water system. The angular frequency of pulsation (*ω*, rad s^−1^) is calculated from the angular velocity of the peristaltic pump (**Eq. 5**). The assessed *α* at the experimental angular velocities and the associated hydrodynamic conditions are presented in Table 1. In each case, the second CIJ had an equal FR to the peristaltic pump.

(5) $$\omega =2\pi \left(\frac{\mathrm{rpm}}{60}\right)$$

**Table 1:** Womersley number and associated parameters.

Angular velocity (rpm)	ω (rad s^−1^)	Flow rate (mL min^−1^)	*α*
24.0	2.5	10	1.5
34.8	3.6	15	1.8
45.0	4.7	20	2.0
47.9⁎	5.0	25	2.2

⁎ Maximum operational value.

Changes in API concentration in the organic solution and solvent were performed afterwards for a more comprehensive exploration of the approach. Experiments were performed in triplicate, and samples were stored at 4 °C protected from light.

#### 2.4.3 Computational fluid dynamics

Three-dimensional flow simulations were performed using the SolidWorks Flow Simulation add-in (v.2024, Dassault Systems, France). Hydrodynamic and mixing-related parameters, including shear stress, volume fraction of the solvent, velocity flow field, vorticity, and turbulent dissipation rate (*ε*), were studied. Adequate global and local mesh refinements were applied in the regions of the computational domain to ensure accurate resolution of flow gradients. Turbulence was modeled using a Reynolds-Averaged Navier-Stokes (RANS) approach with a two-equation eddy-viscosity (k–ε type) closure. Boundary conditions were defined for the CIJ mixers in terms of inlet velocity properties (or volumetric FRs) and outlet static pressure. A rotating region, set at specific angular velocities, was also defined for the peristaltic pump simulations.

#### 2.4.4 Robustness evaluation, design space establishment, and stability assessment

A fractional factorial 2^5–1^ design with resolution V was implemented using the statistical software Minitab 21 (Minitab Inc., USA) to assess the robustness of the HDNP process in terms of HD and PdI. Table 2 shows the factors involved in the study with their denotation as X_1_, X_2_, X_3_, X_4_, and X_5_ for statistical computation and their respective levels (i.e., highest: +1, middle: 0, and lowest: −1). A total of 19 experiments were randomly performed (cf. **Table S4**), consisting of a base design of 16 runs plus a central point (i.e., 0, 0, 0, 0, 0) performed in triplicate to assess reproducibility and curvature of the responses.

**Table 2:** Fractional factorial 2^5–1^ design summary for robustness assessment in HDNP.

Factor	-1	0	+1
X_1_: P407:P188 ratio (stabilizing agent)	0:1	1:1	1:0
X_2_: CIJ #2 ID (mm)	0.4	0.5	0.6
X_3_: DF	5	6	7
X_4_: Stirring rate (rpm)	300	400	500
X_5_: Stirring time (min)	10	12.5	15

ID: internal diameter; DF: dilution factor.

A first-order polynomial equation was obtained for each model, relating the respective response to the input variables, where *b*_*0*_ is the intercept, *b*_*i*_ and *b*_*ij*_ are the regression coefficients for linear and two-way interaction terms, respectively, and *e* accounts for the error (**Eq. 6**).

(6) $$y=b_{0}+\Sigma \;b_{i}X_{i}+\Sigma \;b_{\mathrm{ij}}X_{i}X_{j}+e\;$$

Overlaid contour plots were generated following the identification of significant factors to determine the design space (i.e., a multi-dimensional region enabling a trade-off between CQAs). A representative operating point within this region enabling obtaining DEX NP of 200–250 nm and PdI between 0.15 and 0-25 was selected and experimentally evaluated in triplicate. The obtained results were used to verify the predictive capability of the model and confirm the suitability of the proposed design space. The prepared nanosuspensions were stored at 4 °C protected from light and assessed for HD, PdI, ζP, LE%, and API content for at least two weeks. A kinetic release evaluation was performed at t_0_.

#### 2.4.5 Method applicability

The operating conditions identified for the HDNP of DEX were implemented for PRD nanoprecipitation to evaluate the applicability of the method to low LogP poorly water-soluble drugs.

### 2.5 Statistical analysis

All experiments were performed in triplicate, and data are presented as mean ± standard deviation (SD). Statistical analyses not related to the DoE were performed using GraphPad Prism (v.10.1.0, GraphPad Software, Inc., CA, USA). An unpaired two-tailed Student's *t*-test with Welch's correction was used for comparison between two groups. A two-way repeated-measures analysis of variance (ANOVA) was performed to analyze stability data and drug release kinetics, considering formulation type and time as factors. Tukey's or Bonferroni's post hoc test was done for pairwise comparison as required. Statistical significance was established at *p*-values ˂ 0.05.

## 3 Results and discussion

### 3.1 Hydrodynamically Decoupled Nanoprecipitation proof-of-concept

Confined impinging jet (CIJ) mixing provides optimal conditions for the generation of concentration gradients upon rapid solvent-antisolvent exchange (Hao et al., 2020). However, as observed experimentally, burst nucleation of DEX was not achieved under FNP-based methods with short *t*_*r*_ (cf. **Fig. S1 and S2**). Based on these results, it was hypothesized that increasing the *t*_*r*_ combined with pulsatile shear could enhance nucleation induction under flow-based conditions.

Theoretically, this novel strategy could potentially enhance local supersaturation generation (Qu et al., 2021). However, this should happen prior stabilization to avoid delayed nucleation occurring downstream or at the antisolvent reservoir, as observed in FNP and SNaP. In these approaches, the persistence of supersaturation in the final dispersion might have promoted molecular deposition of DEX onto pre-existing non-stabilized nuclei, ultimately favoring particle growth, aggregation, and precipitation (Saad and Prud'homme, 2016).

Table 3 shows the basic screening plan used to evaluate HDNP feasibility, performed with a total flow rate (TFR) of 100 and 50 mL min^−1^ in the first and second CIJ mixing stages, respectively. As noted, nanoprecipitation was successfully achieved at a DF of 5, with the formed nuclei requiring a P407:DEX mass ratio of 4:1 to be stabilized. This suggests that the large surface area generated, as a result of a high nucleation rate, required rapid steric stabilization by the polymer (Zhao et al., 2023). Similar observations have been reported for other solvent-antisolvent systems, where insufficient stabilizer availability led to particle growth and aggregation despite successful initial nucleation (De Grandi et al., 2022).

**Table 3:** Preliminary screening for HDNP feasibility.

Run	[DEX] (mg mL^−1^)	[P407]:DEX	DF	*Z*-ave (nm)	PdI	[Part.] (part. mL^−1^)
HDNP-1	5	2:1	2.5	Aggregation		
HDNP-2	5	4:1	2.5	Aggregation		
HDNP-3	5	2:1	5	Aggregation		
HDNP-4	5	4:1	5	206 ± 3	0.137 ± 0.034	3.21⁎10^9^ ± 4.5⁎10^7^
HDNP-5⁎	0	1:0	5	202 ± 9	0.313 ± 0.052	1.25⁎10^8^ ± 4.0⁎10^7^

⁎ P407 control; DF: dilution factor.

The required high P407 concentration is also related to the moderate hydrophobicity of DEX, as reported by Jansook et al. (Jansook et al., 2016) This could have limited the interactions between DEX and the hydrophobic PPO core of the tri-BCP. Therefore, the high P407:DEX ratio compensated for the polymer's slow adsorption kinetics onto the surface of newly formed nuclei at the second CIJ stage. The latter has a direct consequence on the effectiveness of the stabilization mechanism by steric hindrance, provided by the hydrophilic PEO chains (Lorscheider et al., 2019).

**Fig. 3:**
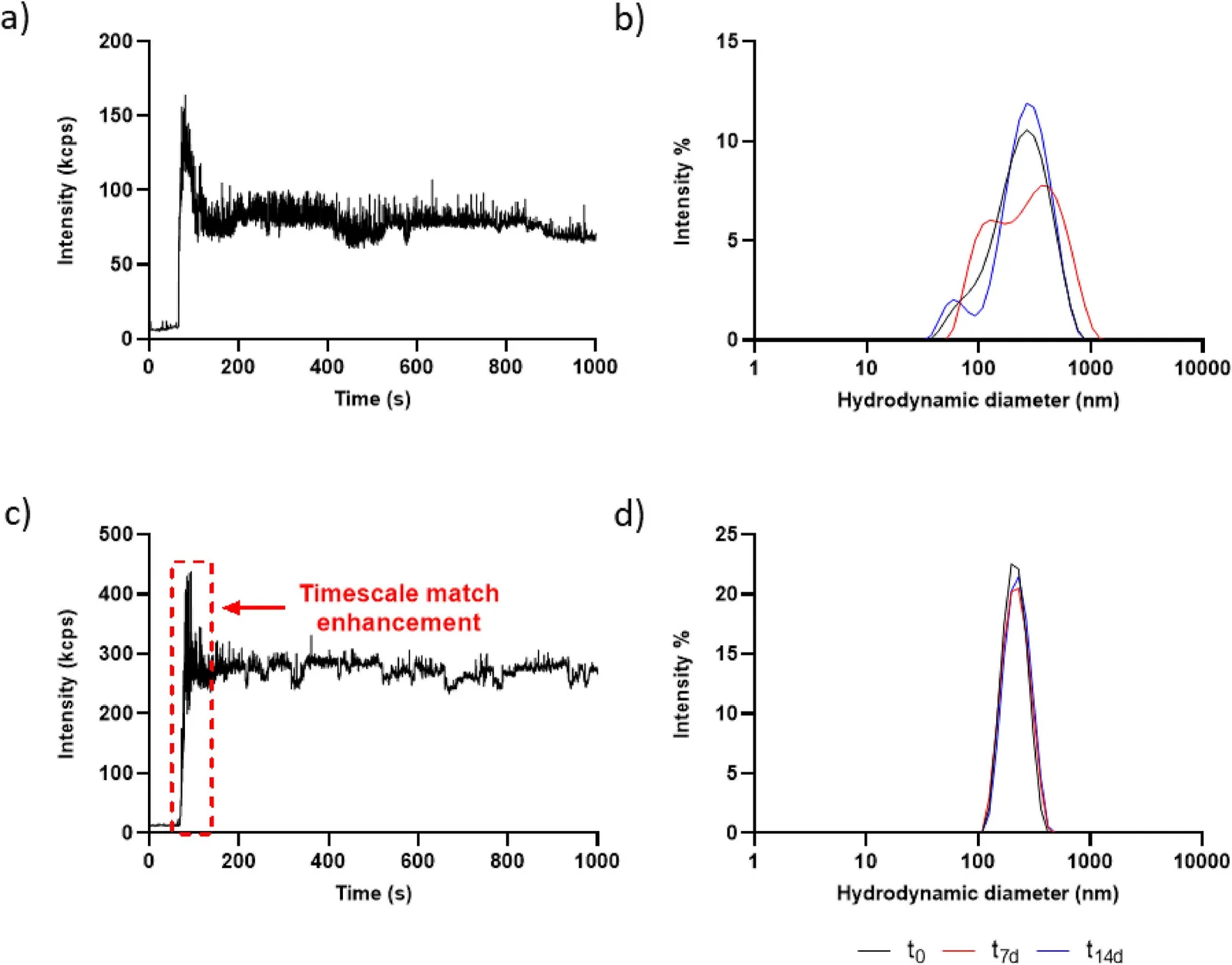
HDNP proof-of-concept: P407 10 mg mL^−1^ control (DF: 5, ethanol content: 5%) showed an increase in count rate after effluent discharging into the antisolvent reservoir **(a)** due to the formation of nano self-assemblies that prevailed up to 14 days (*n* = 3) **(b)**. DEX nucleation and stabilization timescales enhancement (DF: 5, ethanol content: 5%, P407:DEX 4:1) **(c),** leading to a single increase in scattered intensity as regard to the P407 control alone, enabling the formation of monodispersed and stable DEX-NPs up to 14 days (*n* = 3) **(d).**

On the one hand, as presented in Fig. 3a, the control experiment without DEX (HDNP-5) showed a count rate below 60 kcps during the dilution stage, indicating a low number of scattering particles. The resulting NPs, which prevailed up to 14 days (*Z*-ave t_14d_: 214 ± 6 nm and PdI: 0.329 ± 0.048), were consistent with P407 self-association capacity near or above the critical micelle concentration (CMC in H_2_O at 25 °C: 0.7 *w/v*%) due to its amphiphilic triblock structure (Bodratti and Alexandridis, 2018).

On the other hand, HDNP of DEX was the only approach to cause a single and immediate abrupt increase in count rate upon effluent discharge into the antisolvent reservoir. The value remained close to 300 kcps throughout the stirring time (Fig. 3c). Since the count rate is linked to the number of particles and scattered intensity in monodisperse systems, this outcome provided evidence of prior upstream DEX nucleation (Falke and Betzel, 2019). The significantly larger number of scattering nanoparticles obtained compared to the control (*p-*value <0.0001) also indicated the successful nuclei stabilization, leading to the formation of DEX-P407 NPs.

**Fig. 4:**
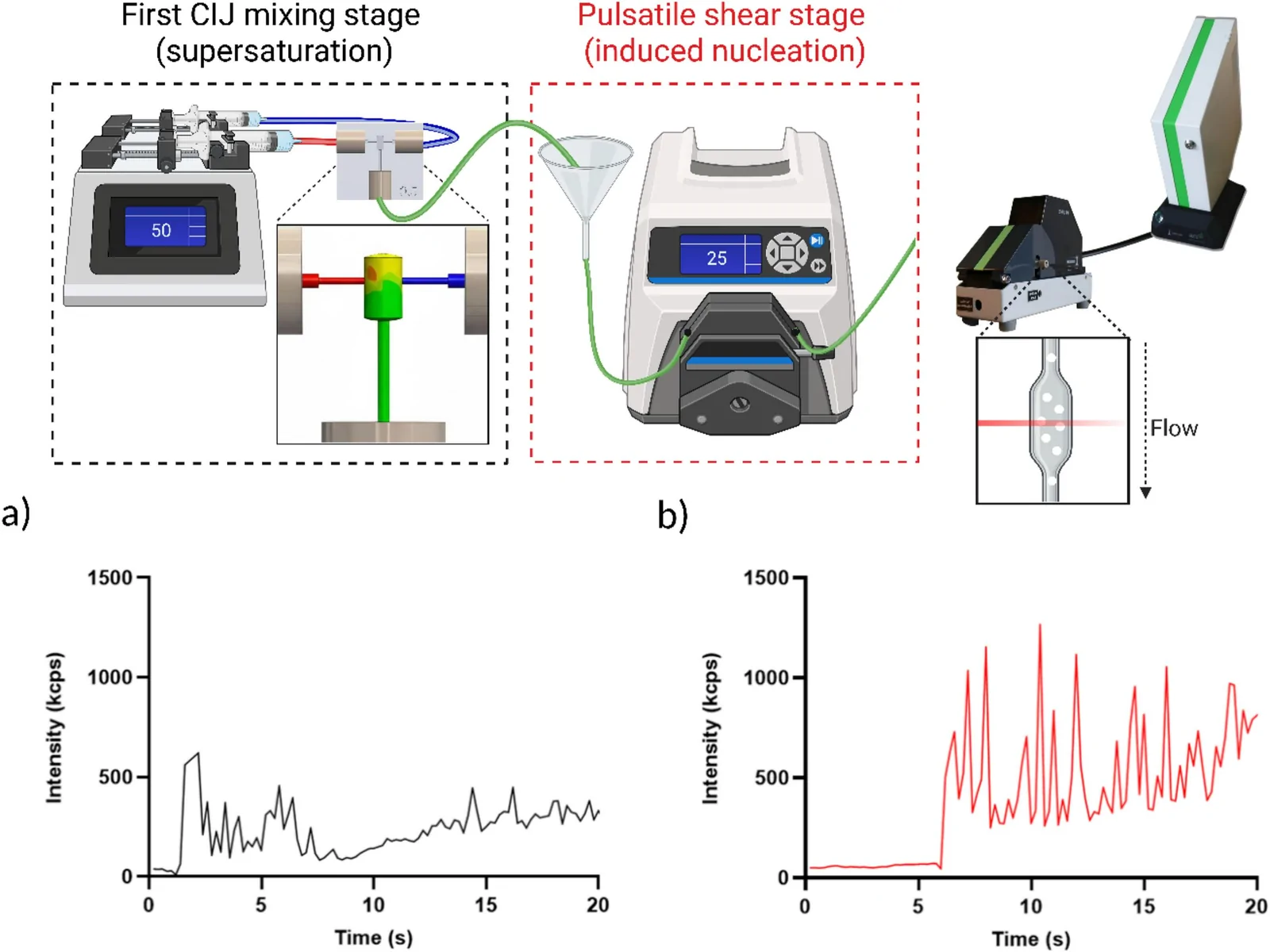
HDNP scattered intensity evaluation per stage using a milli-fluidic in-line DLS-based probe: effluent profile after initial CIJ mixing for supersaturation **(a)** and following pulsatile shear-induced nucleation by the peristaltic pump **(b).**

To further confirm the previous results, the HDNP process was analyzed in terms of the scattered intensity at the first two stages (i.e., CIJ mixing for supersaturation and pulsatile shear-induced nucleation by the peristaltic pump) using a milli-fluidic DLS probe. As seen, a sharp increase in count rate was detected at 1.6 and 6.2 s after injection, respectively. The effluent's profile from the first CIJ mixer (Fig. 4a) corresponds to a solution with transient supersaturation containing prenucleation clusters, as previously observed experimentally by our group (unpublished data). This scattering behavior has also been described by Jawor et al. (Jawor-Baczynska et al., 2013), where changes in DLS-derived signals of nanoscale entities are linked to transient clustering rather than immediate crystal formation. Here, rapid solvent-antisolvent mixing easily overcame the energy barrier to form metastable intermediates, but not for nucleation (Gao et al., 2018).

The second stage involved subjecting the previous mixture to pulsatile flow (Fig. 4b). The two-fold increase in scattered intensity profile indicated a large presence of nuclei readily available for stabilization downstream by P407. This may have resulted from the induced periodic shear stress and higher energy dissipation rates. Both are suitable hydrodynamic conditions for nucleation of organic compounds, as proposed by Yang et al. (Yang et al., 2016) Additionally, based on the experimental hydrodynamic diameters, the second stage demonstrated to provide not only a *t*_*r*_ able to overcome the *t*_*ind*_ but also capable of controlling the nuclei growth timescale to avoid aggregation, as described in SNaP approaches (Caggiano et al., 2023).

Interestingly, no direct evidence tying these pulsatile flow-induced conditions to a non-classical nucleation pathway has been reported. The latter has not been considered either as a stimulus-assisted nanoprecipitation technique (Li et al., 2026), regardless of previous works evidencing the formation of nano- and microparticles in filling pump operations of monoclonal antibodies (Tyagi et al., 2009; Nayak et al., 2011). While these were reported as protein-induced instabilities (Nayak et al., 2011), Her et al. (Her and Carpenter, 2020) further described the influence of tubing mechanical deformation on pressure fluctuation, transient extensional flow, and interfacial stress in these cases.

Therefore, the findings from Fig. 3, Fig. 4 suggest three important aspects worth considering: i) HDNP promoted large nucleation of DEX, following a non-classical pathway under flow-based conditions, by decoupling it temporally and spatially from supersaturation, ii) subsequently, pulsatile shear-induced nuclei were successfully stabilized by P407 in a second CIJ unit, and iii) the absence of DEX delayed nucleation signal in the antisolvent reservoir confirmed proper enhancement of the nucleation and stabilization timescales.

Although the final system is mathematically supersaturated considering the ethanol content (5%, *S*_*0* predicted_: 0.1 mg mL^−1^) and DEX total concentration as determined by HPLC (0.262 ± 0.030 mg mL^−1^), the three previous reasons justify the stability observed for at least 14 days (*Z*-ave t_14d_: 219 ± 4 and PdI: 0.144 ± 0.005). Therefore, the nanosized drug provided a ∼ 2.6-fold increase in the apparent solubility. Additionally, the neutral surface charge of the nanosuspension (ζP: −1.9 ± 0.7 mV) confirmed the stabilizing mechanism governed by steric hindrance (Bodratti and Alexandridis, 2018).

**Fig. 5:**
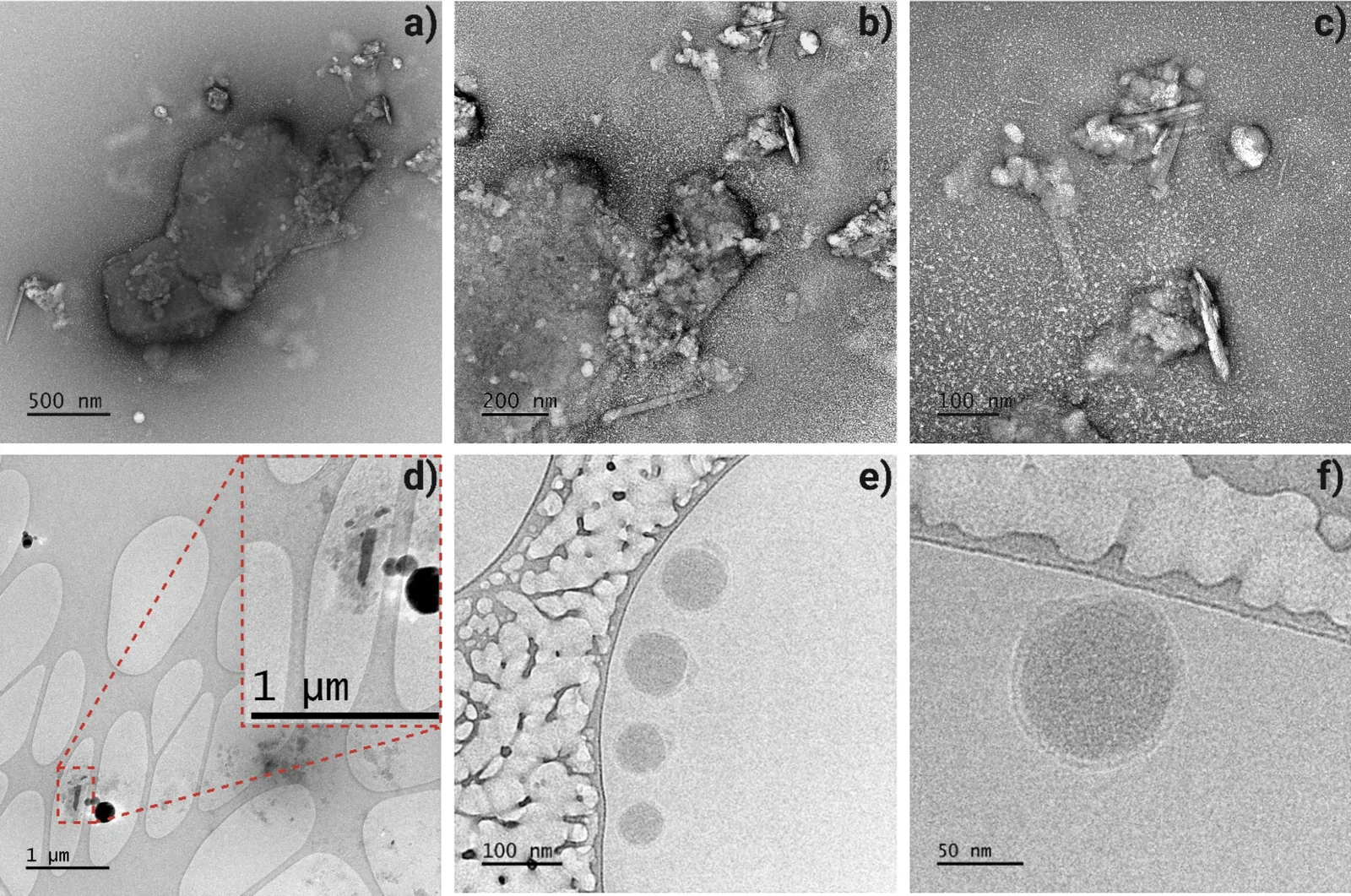
TEM of DEX NPs obtained by HDNP showing the presence of both DEX NCs and amorphous NPs (**a-c**) and cryo-TEM demonstrating the presence of DEX NCs (**d**) and the structure of amorphous NPs as a dense DEX core surrounded by a P407 corona (**e,f**). Both evaluations were performed at equal conditions (DF: 5, ethanol content: 5%, P407:DEX 4:1).

TEM was also performed to confirm the formation of DEX NPs following post-processing. Figs. 5a-c show the presence of a heterogeneous population consisting primarily of spherical NPs (89 ± 18 nm), with a smaller population of elongated structures (141 ± 24 nm). The different electron density of the continuous layer surrounding both type of structures suggests the presence of a thin and uniform P407 coating, resulting from polymer adsorption during the stabilization stage (Castillo Henríquez et al., 2024).

Interestingly, these observations could preliminarily suggest that: i) the spherical NPs could be linked to the amorphous solid state resulting from kinetically limited molecular organization (Saad and Prud'homme, 2016), and ii) the observed anisotropic growth (i.e., directionally dependent) of the elongated structures may be associated with crystal growth typically reported for corticosteroids in solution crystallization (Hadjittofis et al., 2018; Chen et al., 2008). These morphological and potentially solid-state differences may result from variations in local supersaturation and mixing conditions before the stabilization stage. Thus, a premature nucleus in HDNP could appear as a transient amorphous intermediate. The possibility that the latter can potentially undergo an amorphous-to-crystalline transformation during preparation cannot be excluded as a potential mechanism contributing to the observed morphological heterogeneity (Barua et al., 2024). However, TEM alone cannot conclude the solid-state nature of these nanoparticles, therefore requiring further physicochemical characterization.

Moreover, given that the nanosuspensions were not subjected to a purification step, as this could affect their physicochemical stability, non-particulate formulation components (e.g., free stabilizer and residual ethanol) may contribute to the observed background. Additionally, TEM micrographies do not allow for a reliable discrimination between P407-rich NPs or DEX-P407 NPs. The latter is attributed to the intrinsically low electron contrast of the drug and the polymer, as well as the impact of drying artifacts (Chen, 2021; Franken et al., 2017). However, the cryo-TEM evaluation shown in Figs. 5e-f presents more detailed evidence. For instance, a denser DEX core can be appreciated as a darker central region in each NP. Meanwhile, the preserved P407 hydrated PEO chains surround the core, forming a corona-like structure or shell, which is commonly reported for amphiphilic BCP-NPs (Tkachenko et al., 2020).

### 3.2 Womersley number assessment in pulsatile flow

**Fig. 6:**
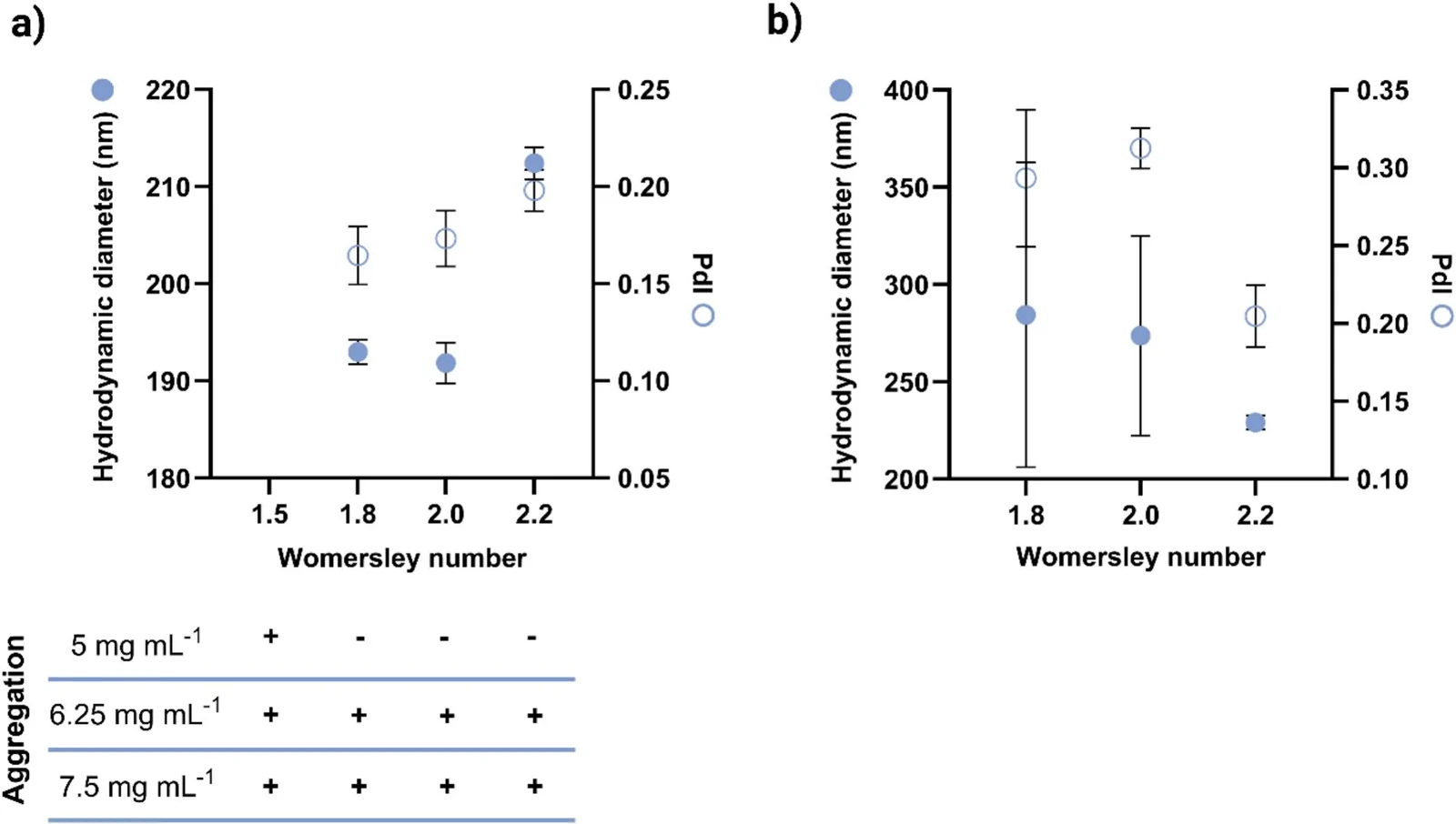
Critical quality attributes of synthesized DEX NP by HDNP as a function of the Womersley number (*α*): HD and PdI evaluation at t_0_ for DEX 5 mg mL^−1^ initial concentration (DF: 5, ethanol content: 5%, P407:DEX 4:1). Inset table shows the initial DEX concentrations that promoted aggregation (+) at the assessed *α***(a)**. HD and PdI evaluation at t_24h_ for the successfully synthesized DEX NPs **(b)**. (Data are presented as mean ± SD, *n* = 3).

To further explore the proposed approach, three different DEX concentrations were assessed along with changes in the Womersley number (*α*, i.e., a dimensionless parameter to characterize pulsatile flow) (Abdelsalam and Vafai, 2017), as presented in Fig. 6. For the 5 mg mL^−1^ DEX initial solution, drug NPs were successfully synthesized at *α* > 1.5 with HDs ranging from 192 to 212 nm and PdI ˂ 0.2 (Fig. 6a). The aggregation observed at 1.5 was due to nucleation taking place in the antisolvent reservoir, given the unsuitable conditions for its generation upstream.

Moreover, while *α* of 2.2 resulted in the largest HD at t_0_, it enabled the greatest physicochemical stability and LE% (68.0 ± 2.9%), with free drug concentration (0.0839 ± 0.0076 mg mL^−1^) approaching the solubility of the system. Conversely, *α* initially yielding smaller NPs resulted in lower LE% (1.8: 59.5 ± 0.5%; 2.0: 62.2 ± 0.5%). These nanosuspensions experienced an increase in both HD and PdI after 24 h storage at 4 °C, as shown in Fig. 6b.

An increase in DEX initial concentration (≥ 25%) at any of the evaluated *α* led to precipitation owing to a temporally and spatially excessive supersaturation (Qu et al., 2021). Thus, 5 mg mL^−1^ could represent the solute load cut-off, and consequently, lower values could, a priori, reduce particle size, as reported before for FNP (Han et al., 2012). Nevertheless, this would in turn impact negatively both the final concentration and the drug loading, considering the solute fraction that remains in solution.

Furthermore, the solvent system directly influences *α* through the viscosity term involved in its calculation. For instance, replacing ethanol with methanol increased *α* owing to the lower viscosity of the resulting system (Maheri et al., 2021). However, despite our previous findings suggesting that a higher *α* enhances the hydrodynamic conditions favorable for HDNP, aggregation was observed across the entire range of explored values when using methanol. This solvent exhibited a stronger solubilizing capacity for DEX than ethanol, which gave rise to higher supersaturation levels when mixed with water. Therefore, under the identified operational DEX initial concentration (5 mg mL^−1^) and the extended *t*_*r*_, precipitation was induced, suggesting that a faster nucleation rate and rapid growth of nuclei made stabilization less effective.

Overall, while the increase in *α* due to process parameters (e.g., angular velocity) resulted in a beneficial effect, its positive effect was outweighed by excessive supersaturation generated by the solvent and/or a high initial drug concentration. Consequently, controlled delayed nucleation in HDNP is conditioned by the interplay between mass transfer, mixing efficiency, and the kinetics of the step (Bałdyga, 2016).

### 3.3 Computational fluid dynamics

Hydrodynamics in the CIJ mixing unit play a critical role in the quality profile of the NPs. Reynolds number (*Re*) ∼90 has been previously reported in classical FNP as the onset threshold for turbulent-like flow in the mixing chamber (Johnson and Prud'homme, 2003b; Devos et al., 2025). However, several authors have acknowledged its limitations in fully characterizing the experimental outcome and the particle formation mechanism. Instead, computational simulations involving parameters that are functions of the local *Re*, such as the RANS approach, have been encouraged for a few decades (Devos et al., 2025; Gavi et al., 2007). Therefore, in order to establish a mechanistic framework for HDNP, CFD was employed to provide a physics-informed perspective on the hydrodynamics governing the approach at the three decoupled stages. Notably, CFD has been used for microfluidics assessment for further understanding of fluid mixing with potential scale-up applications (Bellotti et al., 2024; Bellotti et al., 2026).

**Fig. 7:**
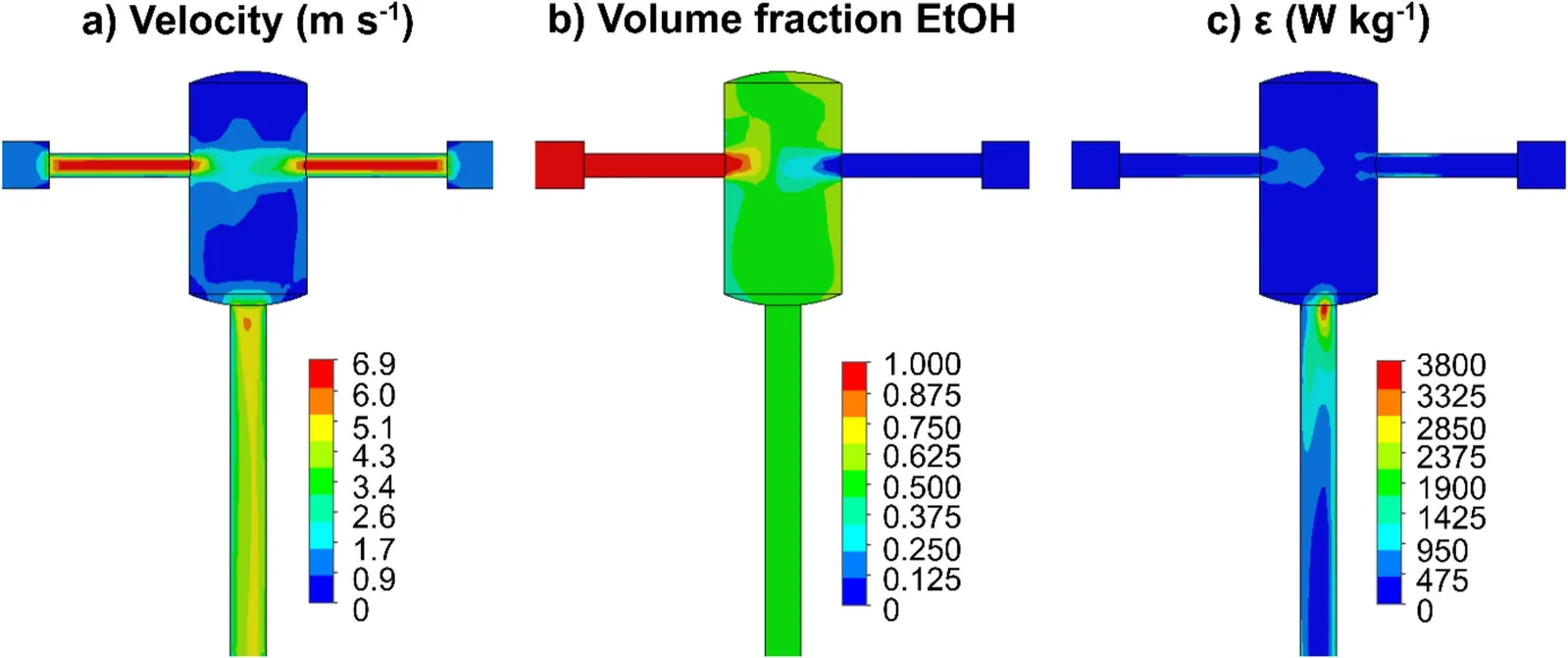
Computational fluid dynamics simulation for the first CIJ mixing stage (supersaturation, TFR: 100 mL min^−1^): velocity flow field **(a)**, evolution of the volume fraction of ethanol **(b)**, and turbulence dissipation, *ε***(c)**. Solvent and antisolvent streams are assigned to the left and right inlets, respectively. The color scale represents the magnitude of each hydrodynamic parameter assessed.

Fig. 7 shows the simulations corresponding to the supersaturation stage at the first CIJ mixer, where the drug solution and antisolvent streams were mixed at a FR of 50 mL min^−1^ each (maximum operational value). The velocity flow field (i.e., fluid velocity variation throughout space and time) displayed intense micromixing conditions at the impinging plane (Fig. 7a). Although a slight flow imbalance could have been presented given the difference in viscosity of the initial solutions (Fonte et al., 2015), suitable high velocities (1.7 to 3.4 m s^−1^) were experienced during the rapid momentum exchange. The latter were indicative of strong convective transport and rapid interpenetration of the solvent and antisolvent jets (Hao et al., 2020).

The efficient micromixing of solvent and antisolvent phases was further confirmed by the rapid dilution of ethanol (Fig. 7b). As the flow moved away from the impingement zone, where the resulting heterogeneous composition (0.25 to 0.75 ethanol volume fraction) generated supersaturation, a more homogenous distribution is observed towards the outlet. Notably, localized regions of higher *ε* (i.e., rate at which turbulent kinetic energy is converted into heat by viscous forces) are concentrated near the impingement zone (475 to 950 W kg^−1^) and the first half of the outlet (Fig. 7c). The latter implies transfer of macroscopic kinetic energy into smaller eddies (i.e., small-scale turbulent vortices enhancing fluid homogenization), which are ultimately, responsible for micromixing, as described by the Kolmogorov energy cascade (Qu et al., 2021).

Notably, the previously described conditions would be expected not only to accelerate supersaturation generation but also to create a favorable environment for nucleation in the case of compounds with high LogP values (El Amri et al., 2025). However, for low LogP drugs like DEX, the first CIJ mixing stage provided a region that acted as the primary micromixing zone, exclusively. As the flow propagated away from the mixing chamber, the reduction in the hydrodynamic parameters indicated further mixing under progressively lower intensities.

**Fig. 8:**
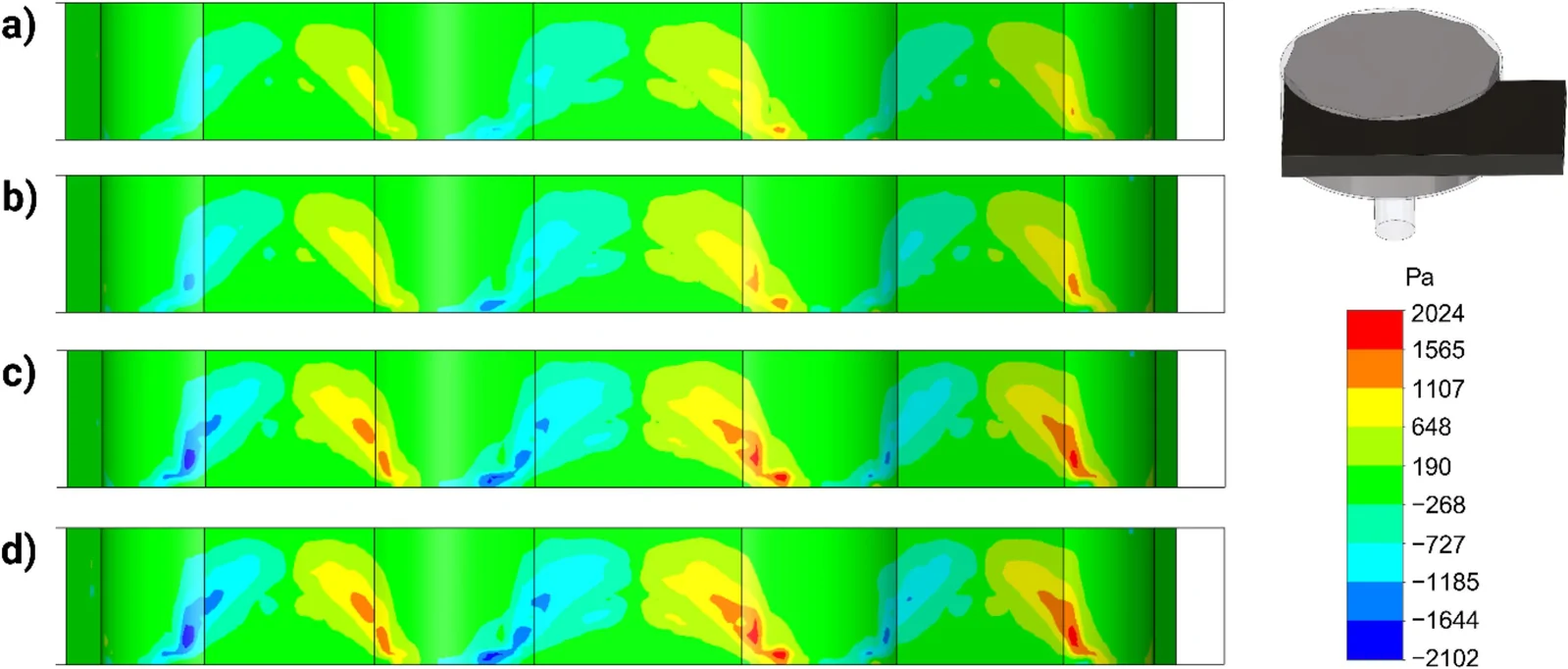
Back view of the computational fluid dynamics simulation for the shear stress (Pa) at the induced nucleation stage using the peristaltic pump at different Womersley numbers (*α*): 1.5 **(a)**, 1.8 **(b)**, 2.0 **(c)**, and 2.2 **(d)**. Flow motion is presented as a progressive positive displacement from right to left.

Moreover, flow simulations of the induced nucleation stage enabled unveiling the hydrodynamic influence of pulsatile flow in terms of the *α*. Fig. 8 presents the shear stress behavior, where positive and negative values were observed in all cases. Positive shear was presented in the regions ahead of the point of closure (i.e., compression zone), while negative values were seen behind it. At *α* of 1.5 and 1.8, the shear fields were relatively moderate, barely exceeding 1000 Pa. However, as the *α* increased up to 2.2, the magnitude and extension of the shear zones duplicated the intensity, revealing a dependency of these high-shear regions on the *α*.

Shear stress simulations strongly suggest local flow reversal and recirculatory behavior as a consequence of the positive displacement caused by the rollers. Contrary to a unidirectional shear field, such cyclic flow deformation may have created local supersaturation regions, which are more prone to nucleation compared with steady-flow conditions (Mudugamuwa et al., 2024). Overall, nucleation in HDNP is likely to be governed by the exposure to these highly stressed regions.

The fluid deformation induced according to the assessed *α* was also confirmed by the velocity flow field, which showed asymmetric profiles (**Fig. S3**). The largest magnitudes resulted from the localized acceleration of the fluid caused by the displacement of the rotating rollers. Vorticity simulations (**Fig. S4**) were also performed to support the shear stress and velocity results. The pseudovector became more pronounced with increasing *α*, leading to the development of secondary flow structures over a larger portion of fluid layers. The latter was particularly notable in the regions between the positive and negative shear fields described above.

**Fig. 9:**
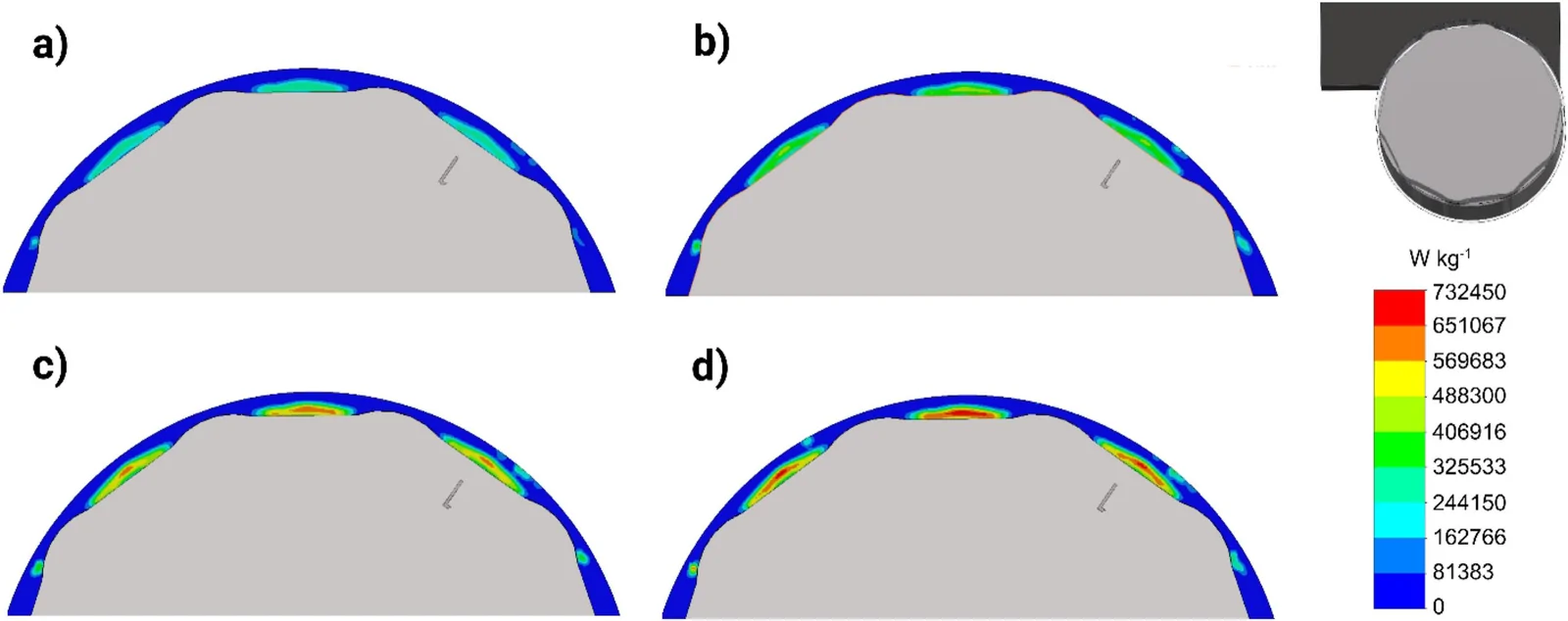
Top view of the computational fluid dynamics simulation for the *ε* (W kg^−1^) at the induced nucleation stage using the peristaltic pump at different Womersley numbers (*α*): 1.5 **(a)**, 1.8 **(b)**, 2.0 **(c)**, and 2.2 **(d).**

Furthermore, as analyzed before for the first stage, *ε* can indicate local mixing intensity and energy transfer at small scales during pulsatile flow as well (Qu et al., 2021). Fig. 9 shows limited regions of elevated dissipation that coincide with zones where hydrodynamic fluctuations were described as functions of shear stress, velocity, and vorticity. In this context, this non-uniform distribution of high-*ε* zones created hydrodynamic hot spots. The existence of such regions provided favorable conditions for shear-induced nucleation (Yang et al., 2016), thus supporting the hypothesis proposed from the experimental observations in Fig. 4b. Additionally, as *α* increased towards 2.2 (Fig. 9d), *ε* reached values above 7*10^5^ W kg^−1^, representing approximately a 184-fold increase compared to the supersaturation stage. The latter implies that a mechanical energy input is capable of generating intense stress fluctuations.

**Fig. 10:**
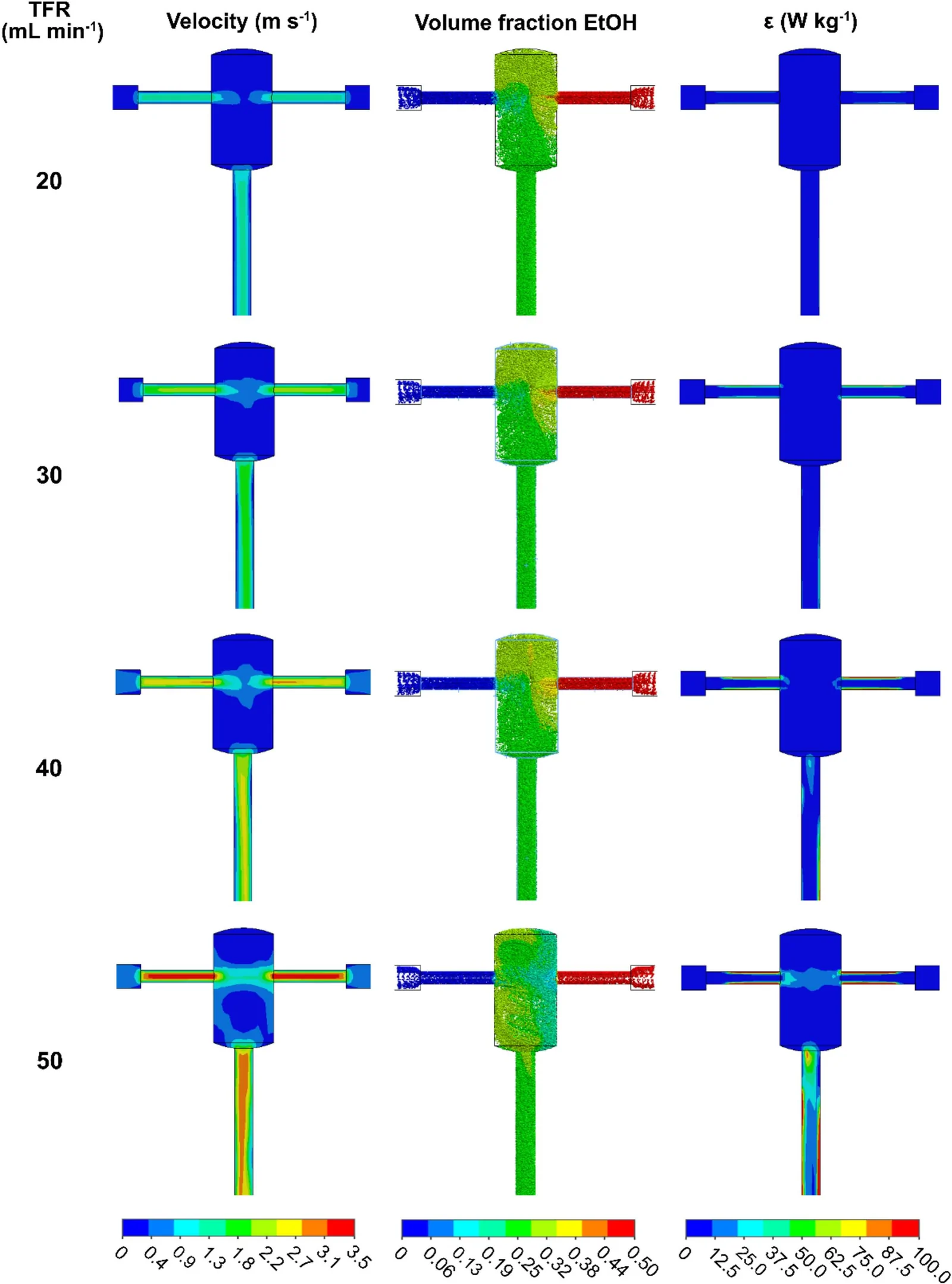
Computational fluid dynamics simulation for the stabilization stage at various TFR (mL min^−1^) for the velocity flow field, volume fraction of ethanol (particle study), and turbulence dissipation, *ε*. Nuclei-containing suspension (50:50 ethanol:water) and aqueous stabilizer solution streams are assigned to the right and left inlets, respectively. The color scale represents the magnitude of each hydrodynamic parameter assessed.

Finally, Fig. 10 shows the CFD results for the velocity flow field, ethanol volume fraction, and *ε* corresponding to the second CIJ mixing or stabilization stage. In here, the nuclei suspension — composed of a 50:50 ethanol:water mixture — and the aqueous P407 solution were mixed at equivalent FRs, ranging from 10 to 25 mL min^−1^. As seen in terms of the velocity field, an optimal impinging plane was achieved at the maximum operational TFR value. This resulted in a region of 1.8 m s^−1^, previously reported within the suitable range for CIJ mixing involving a stabilizing agent stream (Han et al., 2012).

To better illustrate the progressive diffusion of the nuclei suspension and the stabilizer solution, the volume fraction of ethanol is represented as a particle study. Post-stream interpenetration showed particle trajectories establishing a mixed intermediate region at the impingement zone characterized by ethanol fractions between 0.13 and 0.38. However, the region homogeneity was enhanced as the TFR increased. For instance, the higher ethanol concentration that was partially preserved near the nuclei suspension inlet at lower FRs (ethanol volume fraction >0.38) was no longer observed at 50 mL min^−1^ (ethanol volume fraction ˂ 0.19). The more uniform solvent environment around the nuclei potentially reduced local compositional heterogeneities that could have caused particle growth or aggregation (Barua et al., 2024).

Despite the milder hydrodynamic regime in this last stage, the associated higher *ε* (12.5 to 25.0 W kg^−1^) at the highest TFR (50 mL min^−1^) still provided suitable conditions for interaction between the P407 molecules and the nuclei (Hao et al., 2020). Jet momentum promoted a more intensive mixing within the chamber, enabling better polymer adsorption onto the nuclei surface. The latter is consistent with the experimentally observed formation of stable DEX NPs. Therefore, the combined improved homogenization with enhanced local mixing owing to performing at the maximum TFR could have favored fast stabilizer adsorption, limiting particle-particle interactions, and preserving a monodisperse population.

### 3.4 Robustness evaluation and design space establishment

In accordance with the principles of Quality-by-Design (QbD), a DoE approach was implemented (i.e., fractional factorial design 2^5-1^) to evaluate the identified CMAs (e.g., stabilizing agent or P407:P188 ratio) and CPPs (e.g., internal diameter of the CIJ inlets in the stabilization stage, dilution factor, and stirring rate and time) in HDNP 24 h post-production (Chavrier et al., 2026) (cf. **Fig. S5**). After data normalization through Johnson transformation (cf. **Fig. S6**), the multilinear regression showed acceptable goodness of fit for HD and PdI, enabling the models to explain 99.8% and 91.0% of the response, respectively, according to the R^2^-adjusted. This statistical parameter prevents overfitting and provides a more conservative estimation (Wu and Hamada, 2021).

**Fig. 11:**
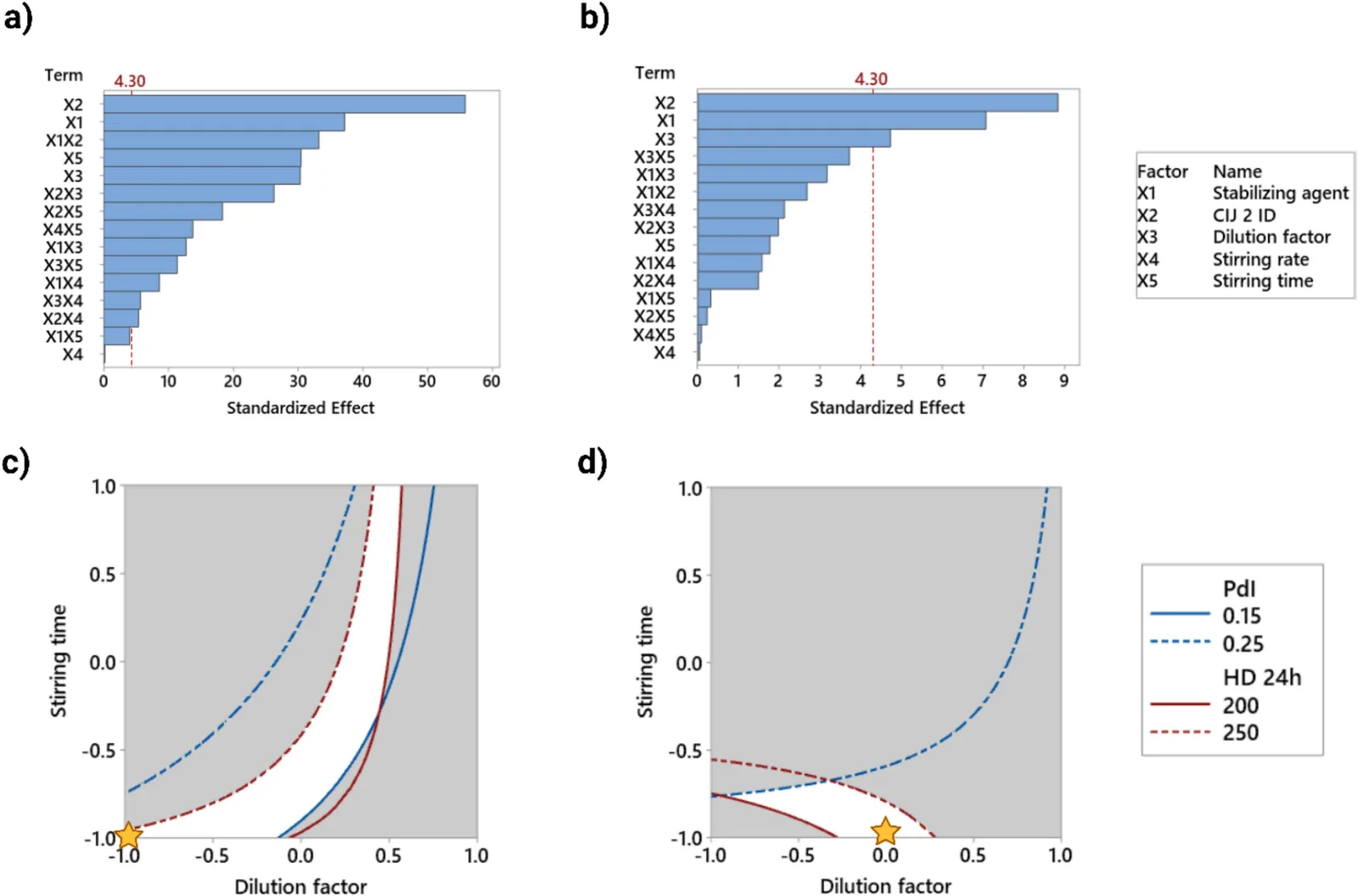
Design of Experiments (DoE) for the evaluation of critical material attributes (CMAs) and critical process parameters (CPPs) in HDNP: Pareto charts of the standardized effects (α = 0.05, *n* = 19) for HD **(a)** and PdI **(b)**. Overlaid contour plots display the design space as the feasible area (white region) for obtaining DEX-NPs within the defined quality specifications for HD at 24 h (red limits: 200–250 nm) and PdI (blue limits: 0.15–0.25) when using P407 (X_1_: +1) **(c)** or P188 (X_1_: −1) **(d)** as stabilizing agent. The star symbol corresponds to a representative operating point in each case (*n* = 3), experimentally evaluated to verify the predictive capability of each model after fixing the internal diameter of the second CIJ mixer (X_2_) and the stirring rate (X_4_) to 0.4 mm (−1) and 400 rpm (0), respectively. (For interpretation of the references to color in this figure legend, the reader is referred to the web version of this article.)

The HDs ranged from 200 to 623 nm when using P407 and from 200 to values above 1000 nm for the experiments employing P188. The former polymer provided, in most cases, reasonably monodisperse preparations, with few exceptions above a PdI of 0.2–0.25. In contrast, P188 led to polydisperse nanosuspensions in almost all the experiments, with a few cases below 0.5. Significant main terms and two-way interactions were identified through this experimental design, as shown in the Pareto charts in Fig. 11a for HD and Fig. 11b for PdI. The high resolution (i.e., V) allowed discarding confusions between these terms with three-way interactions, which are non-significant by default (Wu and Hamada, 2021). Therefore, the significant factors were, indeed, free of confounding, avoiding the execution of additional experiments to discriminate the real critical terms.

On the one hand, HD was affected by all the evaluated terms except the stirring rate and the interaction between the stabilizing agent and the stirring time. On the other hand, PdI appeared to be controlled by a more restricted set of parameters, being the stabilizing agent, the internal diameter of the second CIJ inlets, and the DF. This indicates that nucleation and DEX core growth were more sensitive to process parameters compared to the mechanism involved in population uniformity (Qu et al., 2021). This observation also supported the hypothesis that critical conditions affecting the HD occurred during the rapid mixing and nucleation stages, as described in the CFD study, rather than during bulk mixing.

Moreover, the obtained data were represented as overlaid contour plots to identify suitable regions that fulfill the desired quality specification for both CQAs (i.e., the design space), being a HD range of 200–250 nm and PdI between 0.15 (i.e., operational lower limit) and 0.25. Fig. 11c shows the design space when using P407 (HDNP-DEX/P407) and fixing the conditions to 0.4 mm of internal diameter for the second CIJ inlets and a stirring rate of 400 rpm, as these were identified as optimal for benchtop preparations.

Although the models for HD and PdI suggested that the highest DF (7) led to smaller response values, the plot demonstrates the non-feasibility of said level. Therefore, an operating point, performed in triplicate to test the predictive capacity of the models, involved the lowest DF (5, [DEX]_final_: 0.25 mg mL^−1^) and shortest stirring time (10 min), which was related to smaller DEX NP and monodisperse preparations. The predictive error was 10% for HD (predicted: 219 nm; CI 95%: 213–225 nm; experimental: 241 ± 6 nm) and 3% for PdI (predicted: 0.254; CI 95%: 0.188–0.339; experimental: 0.246 ± 0.023).

Likewise, Fig. 11d presents the design space when using P188 (HDNP-DEX/P188) while fixing the inlet diameter of the second CIJ and the stirring rate as in the previous case. Notably, P188 does not represent an adequate stabilizing agent for DEX due to its hydrophilicity (Bodratti and Alexandridis, 2018). Particle growth was observed after executing the experimental plan as a result of its poor adsorption onto the nuclei during stabilization or drug leakage from the NPs post-preparation (Tao et al., 2019).

Therefore, the operating point required at least a DF equal to 6 ([DEX]_final_: 0.21 mg mL^−1^) in order to contrast the potential remaining supersaturation, and 10 min of stirring time to prevent additional shear stress that could cause further drug leakage. The predictive error was 7% for HD (predicted: 208 nm; CI 95%: 205–211 nm; experimental: 223 ± 7 nm) and 5% for PdI (predicted: 0.274; CI 95%: 0.216–0.347; experimental: 0.261 ± 0.083). In both cases, DoE demonstrated its statistical power for decision-making, providing a rational and systematic approach for improved control over NPs characteristics, fulfilling the defined quality profile (Rampado and Peer, 2023).

Notably, the presented systematic identification of CMAs, CPPs, and the corresponding design space provides a basis for transferring the identified operating conditions to larger (i.e., scale-up) or parallel configurations (i.e., scale-out). This understanding could also enable translating HDNP into a continuous manufacturing approach. However, the latter would require confirming comparable mixing, *t*_*r*_, pressure and shear conditions through dedicated process-control studies to ensure reproducibility, while addressing potential challenges associated with traceability.

### 3.5 Drug release kinetics

DEX in vitro release kinetics from the nanosuspensions were assessed using a dialysis-based method. Cumulative release profiles of each preparation were fitted to an exponential plateau model and compared with that of the micronized drug with the respective polymer, dispersed under identical experimental conditions (cf. **Table S6**). These systems precipitate at room temperature upon mixing due to exceeding the solubility limit. However, their evaluation at 37.5 °C is suitable owing to kinetic stability.

**Fig. 12:**
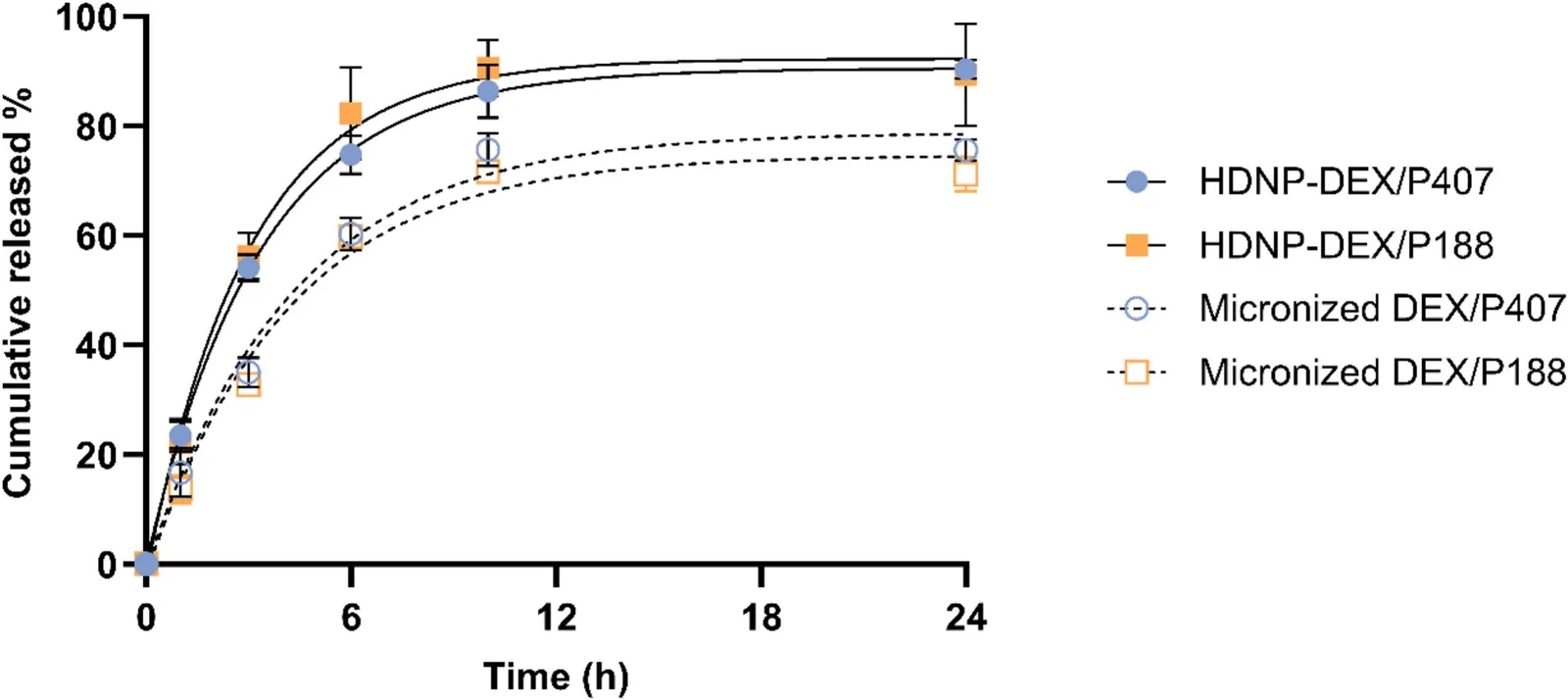
DEX release kinetic profile (non-linear fit) comparison between the nanosuspensions (solid symbols) and the micronized powder dispersions (empty symbols) with P407 (light blue) and P188 (orange) at equal conditions (5% ethanol, P407:DEX 4:1). [DEX] in P407- and P188-based systems was ∼0.25 mg mL^−1^ and 0.21 mg mL^−1^, respectively. Release was performed using a dialysis device in contact with a PBS reservoir (44.5 mL). Samples were incubated at 37.5 °C under orbital shaking (130 rpm) for 24 h. (Data are presented as mean ± SD, *n* = 3). (For interpretation of the references to color in this figure legend, the reader is referred to the web version of this article.)

The non-linear fitting curves demonstrated fast initial release kinetics for all samples (Fig. 12). However, the burst release at 1 h did not show significant differences. At 3, 6, 10, and 24 h, the cumulative released percentages were significantly higher for the nanosuspensions than for the micronized dispersions (cf. **Table S7**). The initial release phase was followed in all cases by a plateau starting around 10 h. The latter was consistent with the fitted maximum release (*Y*_*M*_), where HDNP-based preparations reached ∼90%, while the physical micronized dispersions barely exceeded ∼70%.

The enhanced performance of the HDNP-based preparations is attributed to the large surface-to-volume ratio as a consequence of size reduction, promoting faster dissolution and diffusion across the dialysis membrane (Ma et al., 2022). These results highlight the superior dissolution rate of the nanosized drug compared to the micronized form (*p-value* for P407-based systems: ˂ 0.001; *p-value* for P188-based systems: ˂ 0.001). No differences were found between HDNP-DEX/P407 and HDNP-DEX/P188 (*p-value:* 0.2620) at any time point of the study. The latter preliminary suggests the polymers' exchangeability for dissolution rate enhancement of nanosized DEX (Bodratti and Alexandridis, 2018), at least regarding their effect on the release performance 24 h post-production.

### 3.6 Stability assessment

The prepared HDNP-based nanosuspensions were further assessed in terms of their CQAs during 14 days at 4 °C prior to the emergence of visible aggregates, as presented in Table 4. P407-based preparations exhibited adequate physicochemical stability throughout the evaluation period. HD was maintained within the target for both preparations, with no evidence of particle aggregation, preserving a relatively narrow size distribution as suggested by the PdI values.

**Table 4:** Physicochemical stability of HDNP-DEX/P407 (*n* = 3) and HDNP-DEX/P188 (*n* = 3) during storage at 4 °C.⁎

CQA	HDNP-DEX/P407			HDNP-DEX/P188		
	t_24h_	t_7d_	t_14d_	t_24h_	t_7d_	t_14d_
HD (nm)	241 ± 6	249 ± 4	225 ± 1	223 ± 8	208 ± 3	206 ± 2
PdI	0.246 ± 0.023	0.247 ± 0.004	0.254 ± 0.007	0.261 ± 0.083	0.187 ± 0.021	0.200 ± 0.006
ζP (mV)	−1.2 ± 0.3	−1.8 ± 0.4	−1.5 ± 0.2	−4.3 ± 0.3	−5.9 ± 1.2	−5.7 ± 0.3
LE (%)	77.3 ± 2.1	74.7 ± 1.8	73.4 ± 3.5	69.2 ± 4.5	56.9 ± 8.3	52.0 ± 5.7
DEX content (%)	100.0^⁎^	98.8 ± 1.7	95.9 ± 2.1	100.0*	98.0 ± 1.0	96.3 ± 0.9

⁎ DEX content values were normalized to the initial drug content (mg mL^−1^) at t_24h_: HDNP-DEX/P407 = 0.267 ± 0.016; HDNP-DEX/P188 = 0.208 ± 0.011.

The slightly greater negative ζP value for the P188-based preparation may be attributed to differences in the adsorption and surface packing of the poloxamer chains at the particle-liquid interface. P188's lower molecular weight and reduced PPO content could have resulted in a less compact adsorbed layer at the particle surface compared to P407 (Tuomela et al., 2016). Nevertheless, while HDNP-DEX/P188 exhibited major ζP variations compared to HDNP-DEX/P407, the colloidal behavior of both preparations was preserved due to the known steric hindrance conferred by the polymeric corona (Lorscheider et al., 2019).

Despite the comparable physicochemical stability profiles, surface composition changes experienced by HDNP-DEX/P188 may also be associated with the greater drug leakage observed during storage (Crucho and Barros, 2017). Indeed, the preparations possessed different drug retention capacities. For instance, HDNP-DEX/P407 consistently exhibited higher LE% throughout the study, with the initial value decreasing by ∼4%, while HDNP-DEX/P188 experienced a change of ∼17%. Two-way repeated-measures ANOVA revealed significant effects of formulation type (*p-value*: ˂ 0.01) and storage time (*p-value*: 0.0351) on LE%. Therefore, HDNP-DEX/P407 exhibited a significantly superior drug retention after 14 days (*p value*: ˂ 0.01), as expected.

At last, drug potency remained above 95% throughout the two-week study in both preparations. The latter indicates that the formulation and process conditions did not induce significant degradation of DEX, and overall, demonstrated adequate chemical stability during storage. However, the overall findings suggest that incorporating P407 enhanced DEX stabilization within the nanoparticles during storage compared to P188.

### 3.7 Method applicability

**Fig. 13:**
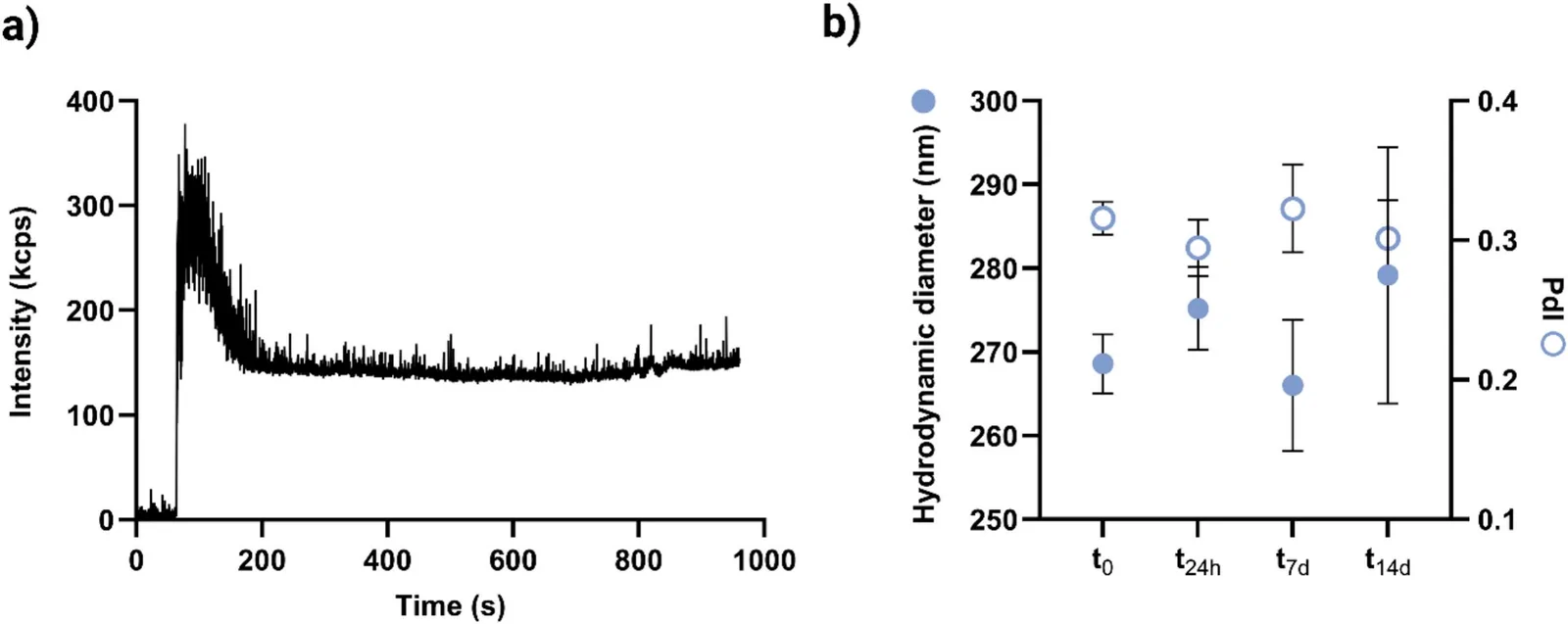
HDNP applicability for PRD: increase in count rate after effluent discharging into the antisolvent reservoir denoting nucleation and stabilization timescales enhancement (DF: 5, ethanol content: 5%, P407:PRD 4:1, [PRD]: 0.25 mg mL^−1^) **(a)** and HD and PdI evolution 14 days post-production **(b).** (Data are presented as mean ± SD, *n* = 3).

Finally, the HDNP approach was applied to a second drug using the operating conditions for DEX and employing P407 for stabilization. PRD, with an even lower LogP value compared to DEX, demonstrated the operational feasibility of the method for low LogP poorly water-soluble drugs. As presented in Fig. 13a, the count rate evolution revealed successful nucleation and stabilization timescales enhancement, evidenced by the single increase in the scattered intensity. Moreover, no significant differences were found for the HD (*p-*value: 0.3542) and neither for the PdI (*p-*value: 0.4662) between t_0_ (269 ± 4 nm; PdI: 0.316 ± 0.012) and t_14d_ (279 ± 15 nm; PdI: 0.301 ± 0.027) (Fig. 13b).

Interestingly, although the HDNP method enabled the nanoprecipitation of PRD, achieving the same quality profile of DEX proved more challenging from a formulation perspective. This behavior can be attributed to its lower LogP value, where the reduced lipophilicity likely limited its affinity for the hydrophobic PPO moieties of P407 (Awuku and Youan, 2026). The adsorption kinetics of the stabilizer onto newly formed nuclei represented a greater limiting step during the early stages of PRD-NP formation compared to DEX. Overall, the results suggest that lower LogP values are associated with increased sensitivity of CQAs to process conditions.

## 4 Conclusions

Hydrodynamically Decoupled Nanoprecipitation (HDNP) was introduced as a novel approach and demonstrated for the nanoprecipitation of a poorly water-soluble corticosteroid with low LogP. The process enabled decoupling supersaturation, nucleation, and stabilization in the nanoprecipitation of DEX, which led mainly to the obtention of spherical nanoparticles. CIJ mixers proved suitable for supersaturation generation and steric stabilization stages. The count rate evolution, monitored by in-line DLS, provided relevant insight for differentiation between pre-nucleation clustering and shear-induced nucleation. Moreover, the developed mechanistic framework involving Womersley number evaluation has been demonstrated to be a useful reference for standardizing pulsatile flow conditions. Further analysis of the hydrodynamic environment through CFD revealed high shear-stress local regions with increased turbulent dissipation during the pulsatile flow stage. The latter combined with the extended *t*_*r*_ explained the observed induced nucleation event.

Furthermore, DoE confirmed the robustness of the HDNP process within the established design space. However, the process exhibited a well-defined operational window for initial drug concentration and solvent composition. DEX concentrations above the experimental range, as well as methanol, were not suitable, leading to aggregation. DoE also enabled the identification of suitable operating conditions for P407- and P188-based formulations. Although no significant difference was observed in release performance between them, further physicochemical characterization revealed HDNP-DEX/P407 superior drug retention and stability during storage. Finally, the successful application of the approach to PRD supports its applicability to low-hydrophobicity, poorly water-soluble corticosteroids. Nevertheless, since DEX and PRD are structurally related, further evaluation of chemically diverse drug classes remains to be performed. Future research will focus on defining the LogP boundaries, understanding the impact of process conditions on the nanoparticle solid-state characteristics, and exploring HDNP scale-up and adaptation to a continuous manufacturing setup.

## CRediT authorship contribution statement

**Luis Castillo-Henríquez:** Writing – review & editing, Writing – original draft, Visualization, Resources, Methodology, Investigation, Funding acquisition, Formal analysis, Data curation, Conceptualization. **Clélia Mathieu:** Writing – review & editing, Visualization, Validation. **Rabah Gahoual:** Writing – review & editing, Methodology. **Dhanang Edy Pratama:** Writing – review & editing, Validation. **Cyrille Richard:** Writing – review & editing, Validation. **Nathalie Mignet:** Writing – review & editing. **José Roberto Vega-Baudrit:** Writing – review & editing, Supervision, Resources. **Yohann Corvis:** Writing – review & editing, Supervision, Project administration.

## Funding

L.C.-H. acknowledges funding from the International Affairs and Foreign Cooperation Office (OAICE) of the University of Costa Rica (contract No. OAICE-13-2022).

## Declaration of competing interest

The authors declare that they have no known competing financial interests or personal relationships that could have appeared to influence the work reported in this paper.

## Data Availability

Data will be made available on request.
