# Supplementary material for: Hydrodynamically decoupled nanoprecipitation (HDNP): A multi-stage strategy for supersaturation, controlled delayed nucleation, and stabilization of low LogP poorly-water soluble drugs
Source: Int J Pharm X. 2026 Aug 30;12:100656. doi: 10.1016/j.ijpx.2026.100656 (PMC13589056; doi:10.1016/j.ijpx.2026.100656)
Supplement: Supplementary file 1 [file mmc1.pdf]

Supporting Information

Hydrodynamically Decoupled Nanoprecipitation (HDNP): A multi-stage strategy for supersaturation, controlled delayed nucleation, and stabilization of low LogP poorly-water soluble drugs

Luis Castillo-Henríquez[1,2,3], Clélia Mathieu[1], Rabah Gahoual[1], Dhanang Edy Pratama[4], Cyrille Richard[1], Nathalie Mignet[1], José Roberto Vega-Baudrit[3], Yohann Corvis[1,*]

Table of contents

Computer-aided design (CAD) of a confined impinging jet (CIJ) mixer 1
Flash Nanoprecipitation (FNP) trials 1
Sequential Flash Nanoprecipitation (SNaP) trials 4
Additional Computational Fluid Dynamics (CFD) 6
Quality Risk Management 7
Design of Experiments (DoE) 9
Drug release kinetics 11

Computer-aided design (CAD) of a confined impinging jet (CIJ) mixer

The CAD of the basic CIJ model designed is presented in **Figure 2**. In order to have stable jets and equal momentum between the two streams, inlet length to jet diameter ratio (L_{in}/d_{in}) was constrained at 6, while the mixing chamber height to diameter ratio (*H/D*) was kept at 0.8, as proposed by Han et al.[1]. The same work proved that an outlet length to exiting jet diameter ratio (L_{out}/d_{out}) ≥ 10 with d_{out} equal to 1.5*d_{in} is enough to provide a pressure drop that allows the mixing chamber to be always filled.

Flash Nanoprecipitation (FNP) trials

FNP was performed as proposed by Johnson and Prud'homme[2]. Absolute anhydrous ethanol was used to dissolve DEX at a concentration of 5 mg mL^{-1}, while water served as the antisolvent

and vehicle for P407 at a concentration of 10 or 20 mg mL^{-1} (P407:DEX 2:1 or 4:1, respectively). A Harvard PHD ultra-syringe pump apparatus (MA, USA) with 20 mL Terumo syringes (Tokyo, Japan) was used to automate the initial mixing at 25 °C in a CIJ unit with 0.5 mm inlet diameter. Each solution was injected at 50 mL min^{-1}, resulting in a TFR of 100 mL min^{-1}. The effluent was directly discharged into an antisolvent reservoir resulting in a dilution factor (DF) of 5, 10, or 15, and subsequently stirred at 500 rpm for 15 min. A Gilson Minipuls3 peristaltic pump (Middleton, WI, USA) with 10 rollers was used at a defined flow rate (FR) to evaluate the impact of shear flow under recirculation. The rationale of the experiments, performed in triplicate, is presented in **Table S1**. **Table S2** presents the theoretical supersaturation conditions generated during CIJ mixing. **Figure S1** shows the count rate monitoring for nucleation evaluation of representative experiments.

Table S1. Exploratory trials for Flash Nanoprecipitation

Run	[DEX] (mg ml^{-1})	[P407] (mg ml^{-1})	DF	Z-ave (nm)	PdI
FNP-1	5	0	0	407 ± 8	0.127 ± 0.060
FNP-2	0	10	0	566 ± 87	0.617 ± 0.061
FNP-3	5	10	0	670 ± 87	0.664 ± 0.055
FNP-4	5	10	5	Aggregation	
FNP-5	5	10	10	Aggregation	
FNP-6	5	20	10	Aggregation	
FNP-7	5	10	15	45 ± 1	0.235 ± 0.010
FNP-8*	0	10	10	15 min post-effluent discharging (stirring)	
				20 ± 17	0.230 ± 0.122
				15 min post-recirculation	
				191 ± 5	0.193 ± 0.025
FNP-9*	5	10	10	After P407 self-assembling (recirculation)	
				204 ± 18	0.305 ± 0.034
				After DEX nucleation (recirculation)	
				Aggregation	

*Performed under recirculation; DF: dilution factor.

Table S2. Theoretical supersaturation conditions generated by CIJ high-energy mixing using an initial 5 mg mL^{-1} DEX organic solution

Stage	DF	Ethanol content (%)	DEX solubility in final system at 25 °C (mg mL^{-1})	[DEX] (mg mL^{-1})	Relative supersaturation (C_t/C^*)
CIJ 1	-	50	5.68	2.5	0.4
Antisolvent reservoir	5	10	0.18	0.5	2.8
	10	5	0.13	0.25	1.9
	15	3.3	0.11	0.17	1.6

DF: dilution factor; C_t: solute concentration; C^*: equilibrium solubility.

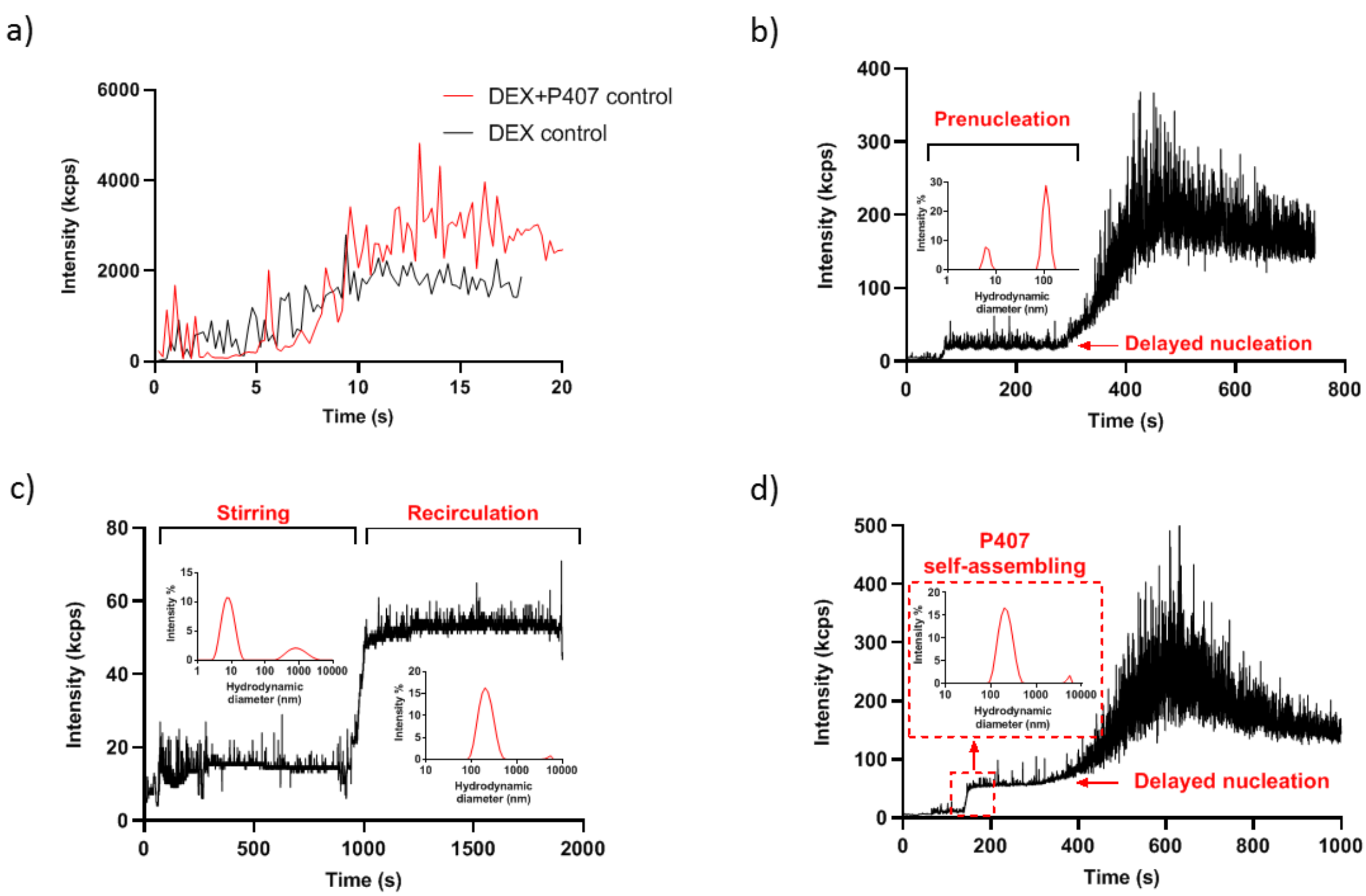

Figure S1. Flash-nanoprecipitation (FNP) exploratory study: DEX induced nucleation by stirring after CIJ-mixing against the antisolvent or a 10 mg mL^{-1} P407 aqueous solution with no further dilution **(a)**. Delayed nucleation behavior when the eluent was discharged into an antisolvent reservoir (DF: 10, final ethanol content: 5%), inset shows the presence of micelles and P407 nano

self-assemblies before nucleation) **(b)**. Count rate evolution for the P407 10 mg mL^{-1} control (DF: 10, final ethanol content: 5%) from stirring (1-16 min; insets show the presence of micelles and P407 self-assemblies) to recirculation through a peristaltic pump at 2.5 mL min^{-1} (16-30 min; insets show the formation of stable P407 nano self-assemblies due to the hydrodynamic shear) **(c)**. DEX-P407 effluent discharged into an antisolvent reservoir volume (DF: 10, final ethanol content: 5%) ongoing recirculation showed mismatched timescales (inset shows the formation of stable P407 nano self-assemblies before nucleation) **(d)** (n = 3)

Sequential Flash Nanoprecipitation (SNaP) trials

SNaP was performed as defined by Caggiano et al.[3] In this approach, the organic solution with DEX at a concentration of 5 mg mL^{-1} was mixed initially with the antisolvent, followed by a minimum increased t_r provided by a 25 cm FEP tube with 0.8 mm of internal diameter. Subsequently, the previous effluent was mixed in a second CIJ unit of 0.5 mm inlet diameter against a 100 mL min^{-1} stream of a 10 mg mL^{-1} P407 aqueous solution (P407:DEX 4:1), using a second Harvard PHD ultra-syringe pump apparatus with a 50 mL Terumo syringe. The resulting final TFR was 200 mL min^{-1} and the DF was 5 (equivalent to DF 10 for FNP). Post-processing conditions were as described for FNP. The rationale of the experiments, performed in triplicate, is presented in **Table S3**. **Figure S2** shows the count rate monitoring for nucleation evaluation of representative experiments.

Table S3. Exploratory trials for Sequential Flash Nanoprecipitation

Run	[DEX] (mg ml^{-1})	[P407] (mg ml^{-1})	DF	Z-ave (nm)	PdI
SNaP-1	0	10	5	86 ± 1	0.104 ± 0.018
SNaP-2	5	10	5	64 ± 1	0.272 ± 0.001
SNaP-3*	5	10	5	After stirring	
				64 ± 1	0.272 ± 0.001
				After recirculation	
				Aggregation	

*Performed under recirculation; DF: dilution factor.

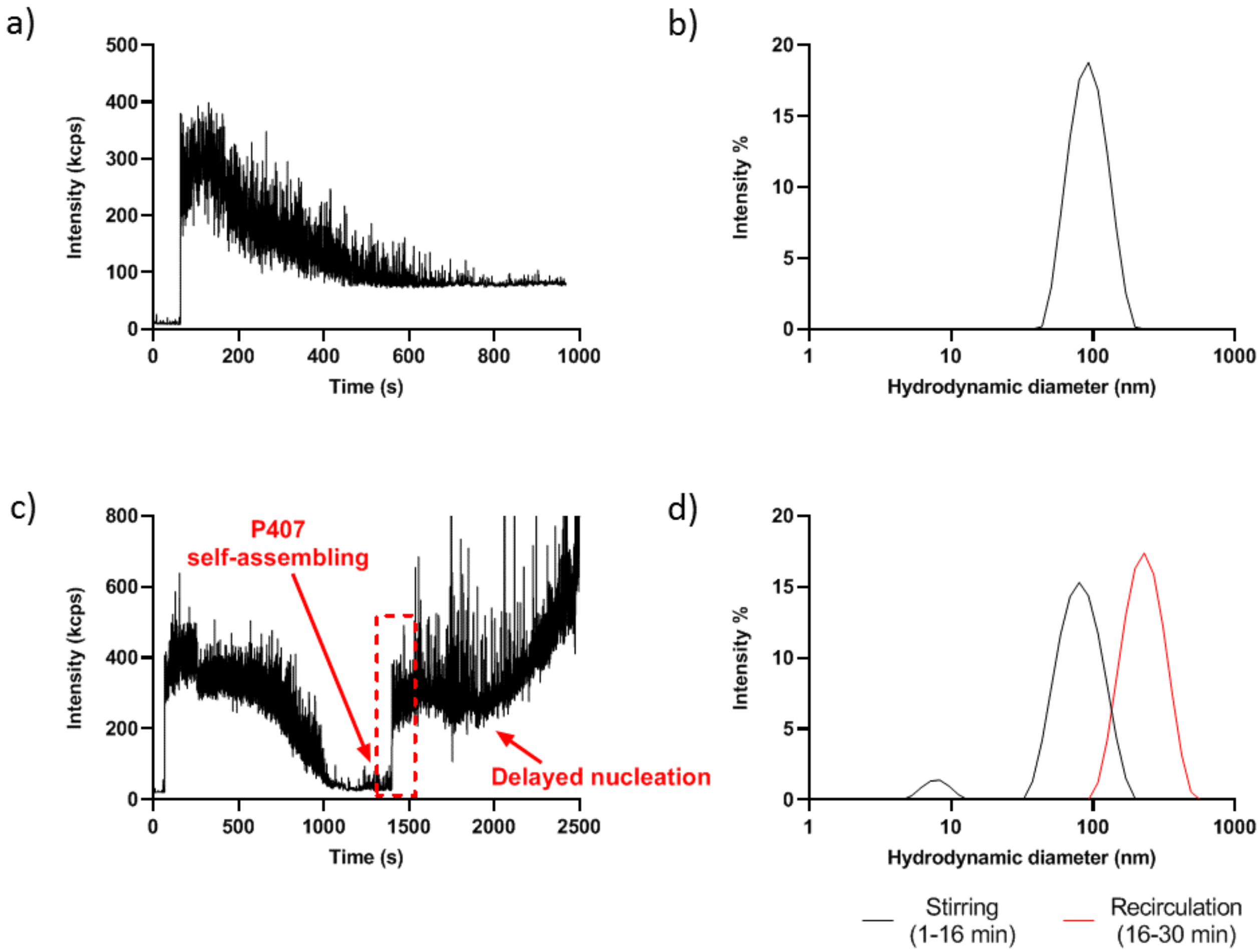

Figure S2. Sequential Flash Nanoprecipitation (SNaP) exploratory study: count rate evolution for the P407 10 mg mL^{-1} control (DF: 5, final ethanol content: 5%) **(a)** showing the presence of unstable nano self-assemblies **(b)**. Unsuccessful nucleation and stabilization of DEX (DF: 5, final ethanol content: 5%) after sequential CIJ mixing leading to delayed nucleation due to recirculation **(c)**, preceded by polymer self-assembling **(c,d)**. Hydrodynamic diameter determinations were done at the end of the stirring stage and one minute after recirculation (n = 3)

Additional Computational Fluid Dynamics (CFD)

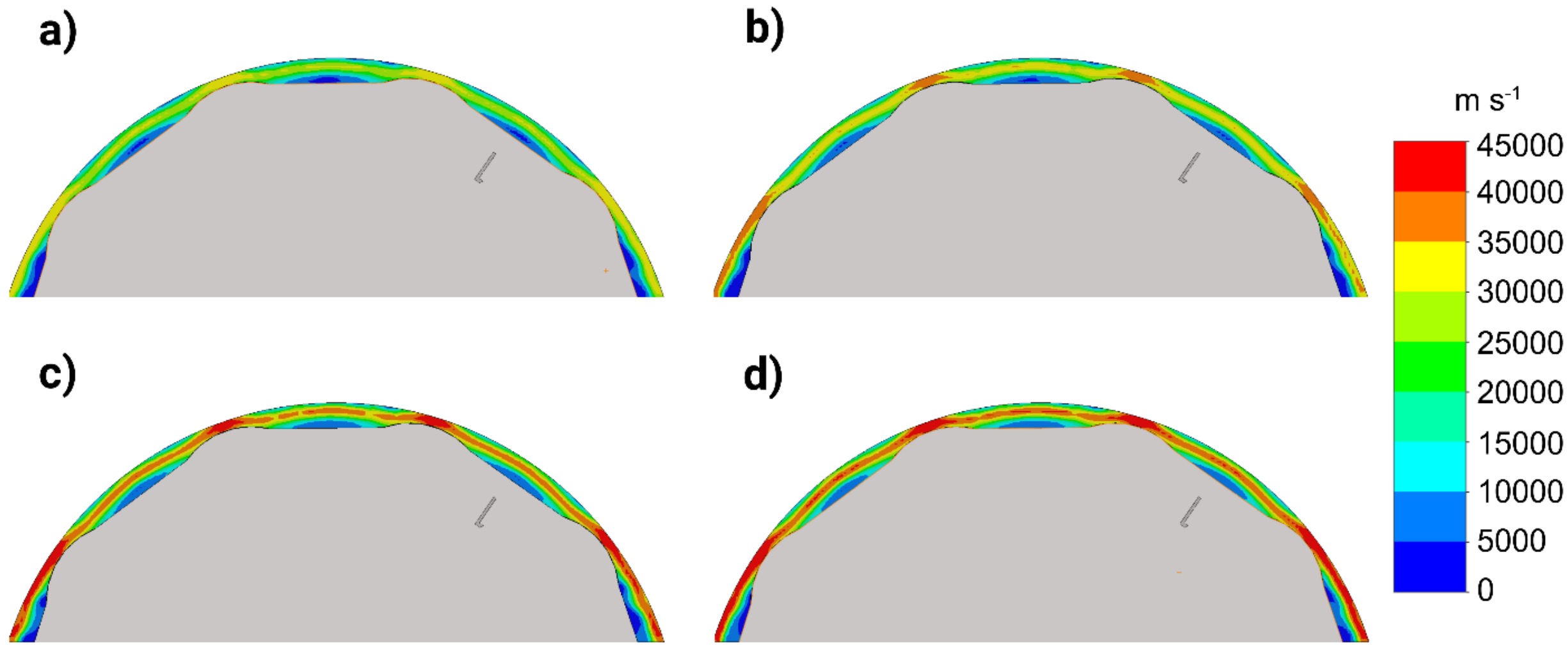

Figure S3. Top view of the computational fluid dynamic simulation for the velocity flow field (m s^{-1}) at the induced nucleation stage using the peristaltic pump at different Womersley numbers (α): 1.5 **(a)**, 1.8 **(b)**, 2.0 **(c)**, and 2.2 **(d)**

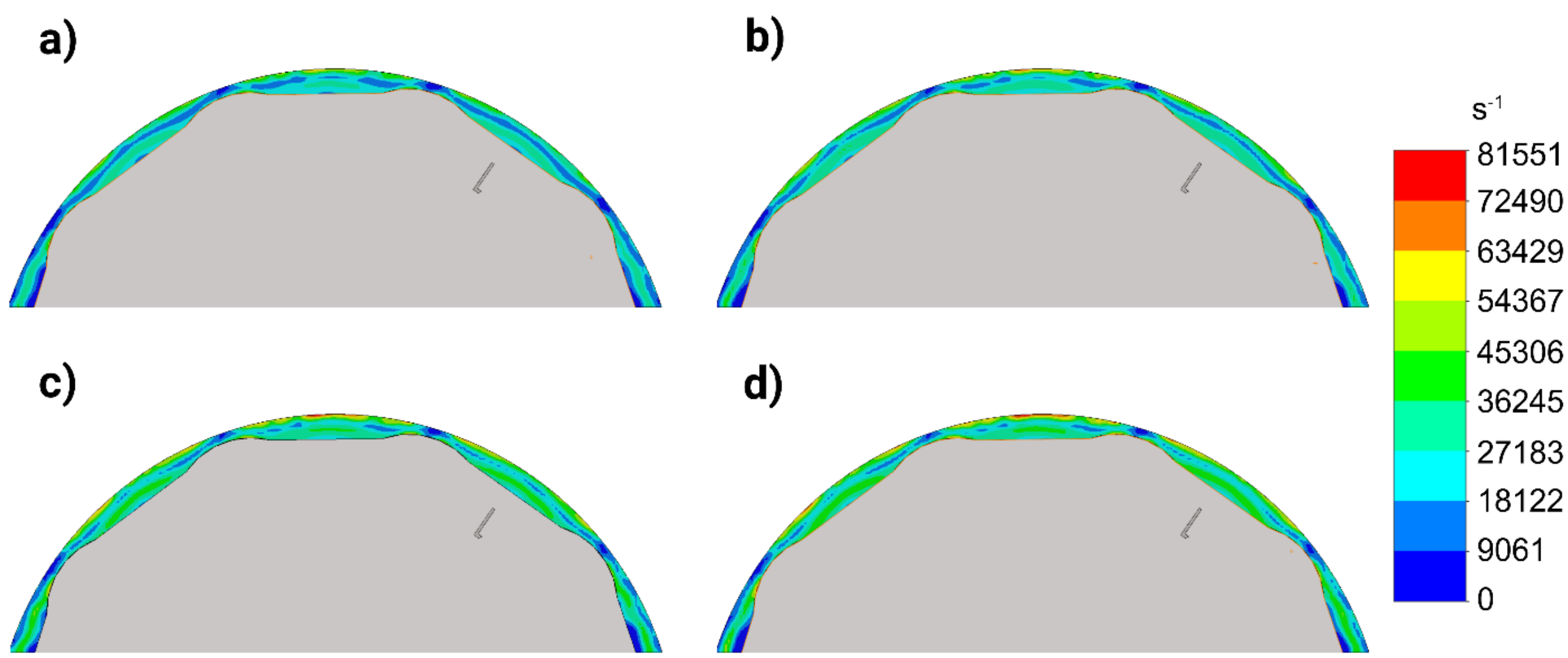

Figure S4. Top view of the computational fluid dynamic simulation for vorticity (s^{-1}) at the induced nucleation stage using the peristaltic pump at different Womersley numbers (α): 1.5 **(a)**, 1.8 **(b)**, 2.0 **(c)**, and 2.2 **(d)**

Quality Risk Management

A formal risk assessment through the Failure Mode and Effects Analysis (FMEA) was done. Critical material attributes (CMAs) and critical process parameters (CPPs) were identified and given a risk priority number (RPN) depending on the impacted critical quality attribute (CQA). RPNs were calculated as shown in **Equation S1** on the basis of severity (*S*, i.e., impact on a CQA), occurrence (*O*, i.e., probability of affecting a CQA), and detectability (*D*, i.e., likeliness of recognizing the failure or quality variation because of the factor) according to the evidence from the consulted literature.

$$RPN = S * O * D \qquad (S1)$$

Each term of the RPM was categorized in five levels:

- *Severity:* from 1 (minimum impact) to 5 (critical impact).
- *Occurrence* (based on the number of papers reporting a significant effect)*:* 1 (1 - 3), 2 (4 – 7), 3 (8 – 11), 4 (12 – 16), and 5 (> 16).
- *Detectability:* from 1 (easily detectable) to 5 (hardly detectable).

Low- (RPN ≤ 26), middle- (27 ≤ RPN < 64) and high-risk factors (RPN ≥ 64) were further analyzed concerning their relationship to HDNP and the selected ones were evaluated by DoE.

Figure S5 shows the impact of the identified CMAs and CPPs on the CQAs, where the corresponding RPN values are represented as a heat map. For the purpose of defining a DoE, particle size and PdI were selected as the reference CQAs due to their ease of evaluation. The stabilizing agent and the antisolvent reservoir were found to have a significant impact on these variables. Therefore, both were included as DoE factors. Other CMAs and CPPs with medium or high RPN values were not considered due to operational constraints or previously established operating conditions. Conversely, the absence of evidence in the consulted literature regarding the CIJ inlet diameter, stirring rate, and stirring time motivated their inclusion in the DoE study to assess their potential impact on the quality profile of the nanosuspensions.

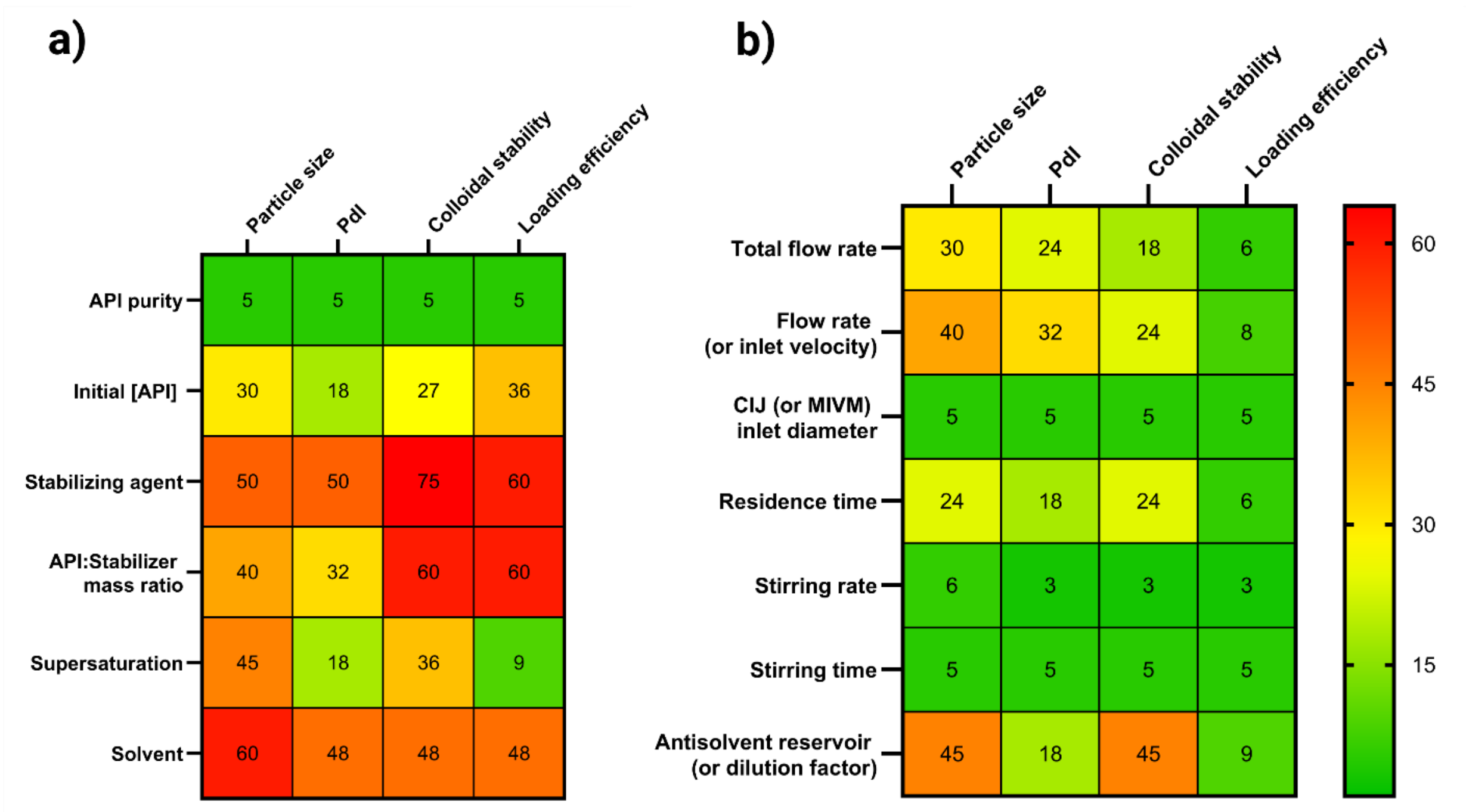

Figure S5. Heat map analysis based on the risk priority number (RPN) of each critical material attribute (CMA) **(a)** and critical process parameter (CPP) **(b)** on the critical quality attributes (CQAs). Low-risk (green gradient): 1-26, middle-risk (yellow gradient): 27-63, high-risk (red gradient): ≥ 64

Design of Experiments (DoE)

Table S4. Design of Experiments uncoded plan with results 24 h post-preparation

Std order	Stabilizer	CIJ 2 ID	DF	Stirring rate (rpm)	Stirring time (min)	HD (nm)	PdI
10	P407	0.4	5	500	15	516 ± 28	0.418 ± 0.031
15	P188	0.6	7	500	10	Aggregation	
12	P407	0.6	5	500	10	254 ± 12	0.349 ± 0.163
3	P188	0.6	5	300	10	901 ± 23	0.852 ± 0.111
5	P188	0.4	7	300	10	200 ± 6	0.252 ± 0.040
1	P188	0.4	5	300	15	757 ± 160	0.686 ± 0.202
16	P407	0.6	7	500	15	232 ± 2	0.285 ± 0.040
13	P188	0.4	7	500	15	212 ± 3	0.190 ± 0.023
8	P407	0.6	7	300	10	220 ± 4	0.197 ± 0.026
17	50:50	0.5	6	400	12.5	276 ± 5	0.468 ± 0.068
11	P188	0.6	5	500	15	Aggregation	
19	50:50	0.5	6	400	12.5	288 ± 13	0.427 ± 0.021
2	P407	0.4	5	300	10	222 ± 4	0.234 ± 0.006
14	P407	0.4	7	500	10	200 ± 16	0.147 ± 0.010
18	50:50	0.5	6	400	12.5	287 ± 14	0.251 ± 0.008
7	P188	0.6	7	300	15	Aggregation	
6	P407	0.4	7	300	15	213 ± 4	0.136 ± 0.024
9	P188	0.4	5	500	10	220 ± 5	0.176 ± 0.024
4	P407	0.6	5	300	15	623 ± 35	0.620 ± 0.221

ID: internal diameter; DF: dilution factor; HD: hydrodynamic diameter; PdI: polydispersity index.

Table S5. Factorial regression for hydrodynamic diameter at t_{24h}

Term	Y_1: Hydrodynamic diameter at t_{24h}	Y_2: PdI at t_{24h}
R^2 %	99.98	99.00
R^2 adj %	99.80	91.00
Predictive linear equation	$Y_1 = -0.0408 - 0.4303 X_1 + 0.6435 X_2 - 0.3510 X_3 + 0.0028 X_4 + 0.3526 X_5 - 0.3842 X_1X_2 - 0.1475 X_1X_3 - 0.1000 X_1X_4 + 0.0472 X_1X_5 + 0.3044 X_2X_3 + 0.0628 X_2X_4 - 0.2123 X_2X_5 + 0.0662 X_3X_4 - 0.1316 X_3X_5 - 0.1596 X_4X_5 - 0.0582$ Ct Pt	$Y_2 = -0.0381 - 0.5402 X_1 + 0.6504 X_2 - 0.3481 X_3 + 0.0034 X_4 + 0.1303 X_5 - 0.1974 X_1X_2 - 0.2333 X_1X_3 + 0.1162 X_1X_4 - 0.0236 X_1X_5 + 0.1455 X_2X_3 + 0.1095 X_2X_4 - 0.0169 X_2X_5 + 0.1566 X_3X_4 - 0.2739 X_3X_5 - 0.0067 X_4X_5 - 0.0930$ Ct Pt

a)

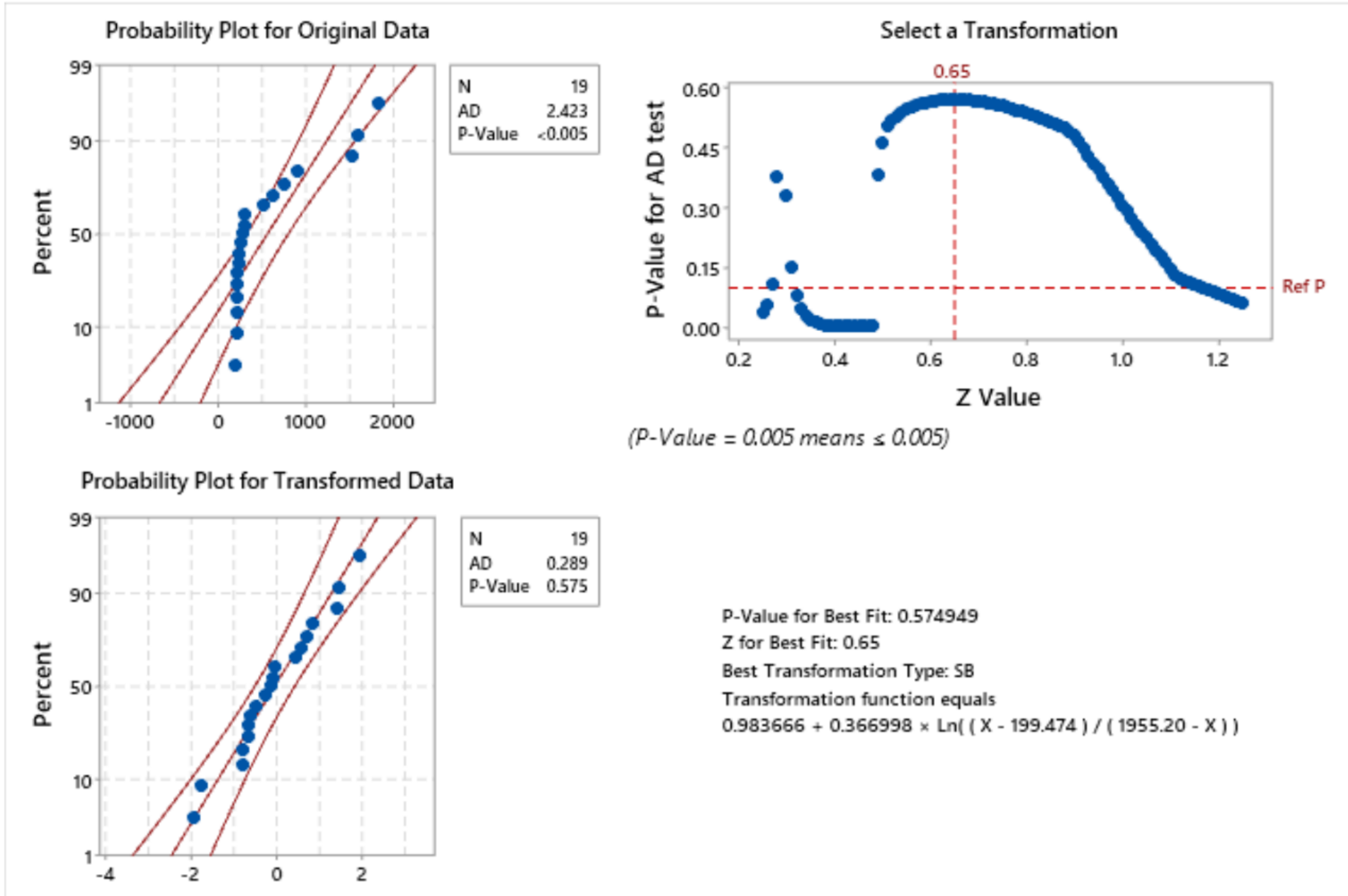

b)

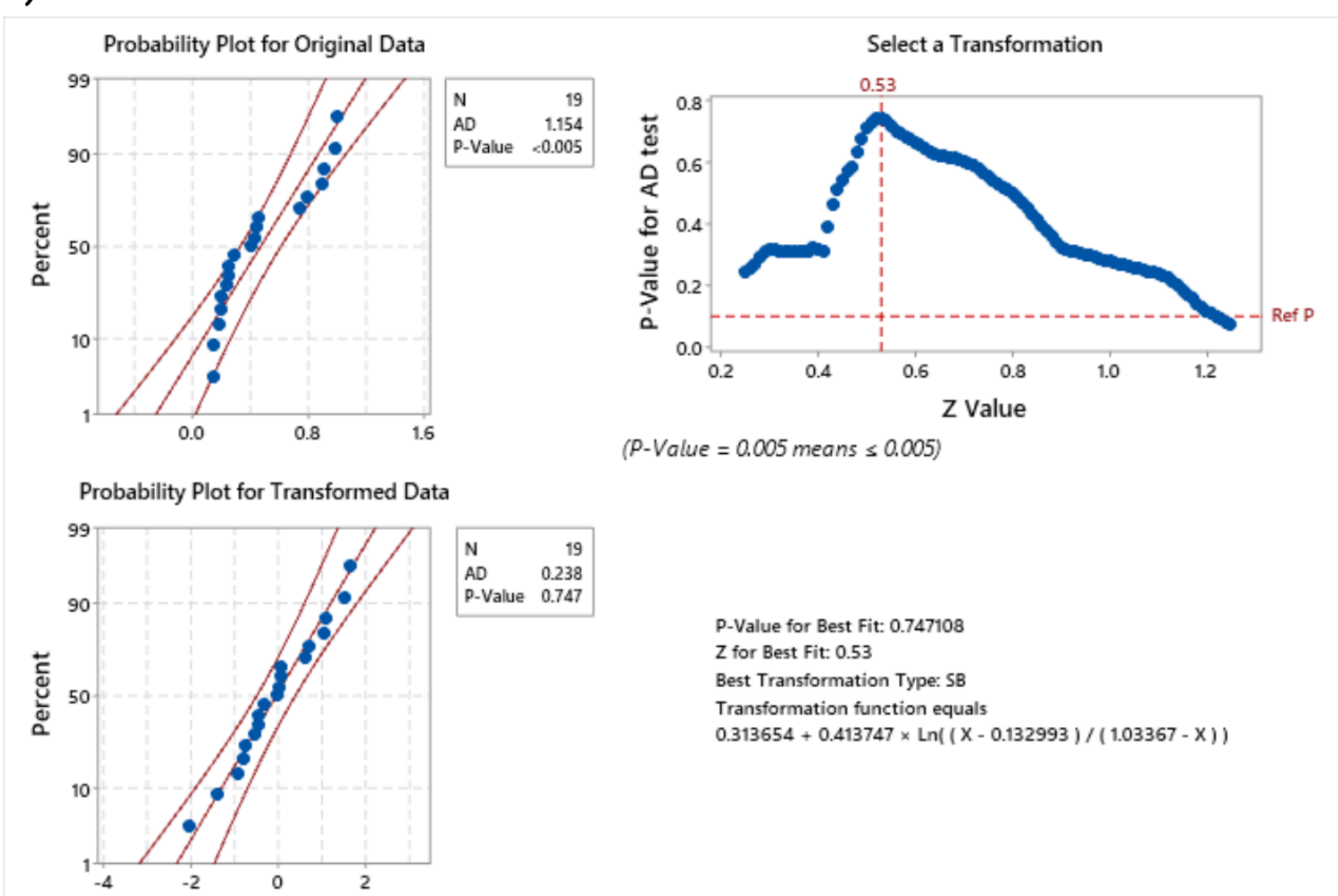

Figure S6. Johnson transformation for hydrodynamic diameter **(a)** and PdI **(b)**

Drug release kinetics

Table S6. Exponential plateau model (n = 3 per treatment)

Best-fit values	HDNP-DEX/P407	Micronized DEX/P407	HDNP-DEX/P188	Micronized DEX/P188
Y_M (%)	90.51	78.84	92.30	74.70
Y_0 (%)	0.07811	-0.7548	-1.804	-1.910
k (h^{-1})	0.2996	0.2335	0.3308	0.2417
R^2	0.9945	0.9837	0.9762	0.9833

Table S7. Statistical comparisons (*p*-values)

Two-way ANOVA				
Source of variation		P407-based preparations	P188-based preparations	HDNP-based preparations
Time		< 0.0001	< 0.0001	< 0.0001
Formulation		0.0009	0.0004	0.2620
Time * Formulation		0.0218	0.0752	0.4949
Bonferroni’s test for multiple comparisons				
Time (h)	1	0.0885	0.1771	> 0.9999
	3	< 0.0001	<0.0001	> 0.9999
	6	< 0.0001	<0.0001	0.3487
	10	0.0024	0.0003	> 0.9999
	24	< 0.0001	0.0004	> 0.9999

References

[1] J. Han, Z. Zhu, H. Qian, A. R. Wohl, C. J. Beaman, T. R. Hoye, C. W. Macosko, *Journal of Pharmaceutical Sciences* **2012**, *101*, 4018.

[2] B. K. Johnson, R. K. Prud’homme, *AIChE Journal* **2003**, *49*, 2264.

[3] N. J. Caggiano, S. K. Nayagam, L. Z. Wang, B. K. Wilson, P. Lewis, S. Jahangir, R. D. Priestley, R. K. Prud’homme, K. D. Ristroph, *International Journal of Pharmaceutics* **2023**, *640*, 122985.
